# 4-Chloroisocoumarins as Chlamydial Protease Inhibitors and Anti-Chlamydial Agents

**DOI:** 10.3390/molecules29071519

**Published:** 2024-03-28

**Authors:** Matthew J. A. Phillips, Wilhelmina M. Huston, Andrew M. McDonagh, Tristan Rawling

**Affiliations:** 1 School of Mathematical and Physical Sciences, Faculty of Science, University of Technology Sydney, Sydney, NSW 2007, Australia; matthew.phillips-1@uts.edu.au; 2 School of Life Sciences, Faculty of Science, University of Technology Sydney, Sydney, NSW 2007, Australia; wilhelmina.huston@uts.edu.au

**Keywords:** anti-chlamydial, protease inhibitor, isocoumarin

## Abstract

4-Chloroisocoumarin compounds have broad inhibitory properties against serine proteases. Here, we show that selected 3-alkoxy-4-chloroisocoumarins preferentially inhibit the activity of the conserved serine protease High-temperature requirement A of *Chlamydia trachomatis*. The synthesis of a new series of isocoumarin-based scaffolds has been developed, and their anti-chlamydial properties were investigated. The structure of the alkoxy substituent was found to influence the potency of the compounds against High-temperature requirement A, and modifications to the C-7 position of the 3-alkoxy-4-chloroisocoumarin structure attenuate anti-chlamydial properties.

## 1. Introduction

*Chlamydia trachomatis* is a gram-negative bacteria and common sexually transmissible infection (STI). Chlamydia is the most-frequently diagnosed bacterial STI, with the majority of diagnoses for both men and women being from 15 to 24 years of age [1]. Persistent infection can cause infertility in men and women as well as pelvic inflammatory disease (PID) and ectopic pregnancy [2].

Current treatment regimens use Azithromycin or Doxycycline. Azithromycin can and has previously been used to treat *Neisseria gonorrhoeae* and *Mycoplasma genitalium*, which are often present as a co-infection with *C. trachomatis*. Each of these infections may present asymptomatically, and with increasing rates of resistance to Azithromycin in *Neisseria gonorrhoeae*, there are concerns with overreliance in its use as a front-line anti-chlamydial therapy. Recent reports recommend the use of Ceftriaxone preferentially to Azithromycin in concurrent chlamydial infections [3]. The contraindication of Doxycycline for use with pregnant women restricts its potential as a replacement front-line therapy [4]. Therefore, new drugs with novel modes of action are needed.

The serine protease **H**igh-**t**emperature **r**equirement **A** (HtrA) has become an attractive anti-microbial target. The development of small molecules targeting HtrA in *Eschericia coli* (DegS) [5] and *Helicobacter pylori* [6] has demonstrated the potential of HtrA inhibitors as a valid antibacterial target in vitro. The HtrA of *C. trachomatis* (CtHtrA) functions as a proteolytic enzyme regulating homoeostasis, and it chaperones membrane proteins through the periplasm [7]. CtHtrA likely has a critical role during stress conditions [8], and exposure of *C. trachomatis* to CtHtrA inhibitors during the mid-replicative phase of the development cycle is lethal [9]. These inhibitors are effective as they prevent both functions of HtrA and restrict bacterial replication, preventing the production of further progeny.

**Figure 1:**
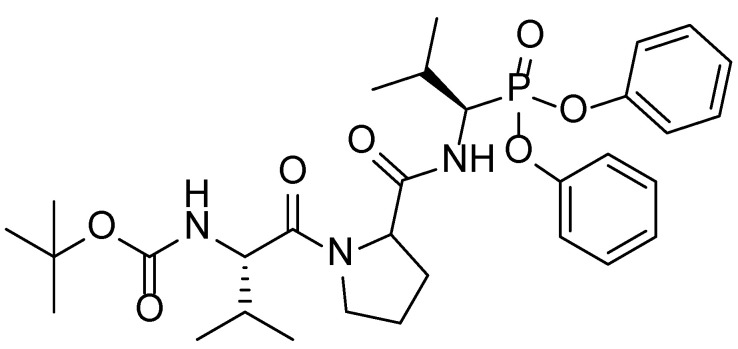
Structure of JO146.

The development of CtHtrA inhibitors has produced diphenylphosphonate peptide-based inhibitors such as JO146 (Figure 1). JO146 features a tripeptidomimetic backbone with a terminal N-Boc capping group and an electrophilic diphenylphosphonate headgroup that reacts with the serine protease to form a reversible covalent complex with CtHtrA (IC_50_ = 12 µM) [9]. JO146 exhibits anti-chlamydial properties against *C. trachomatis* and other chlamydial species with minimal cytotoxic properties against mammalian cell lines including HEp-2 cells, McCoy B cells and ex vivo Koala mononuclear blood cells [9]. Similar structures with modifications to the N-terminal capping group retain their anti-chlamydial activity [10]. However, their inhibitory activity against CtHtrA has not been investigated.

Successive optimization studies have investigated the effects of replacing various amino acids within the JO146 structure, and substitution on the pyrrolidine ring, with some improvement to the inhibitory activity without loss of anti-chlamydial properties [11,12]. However, off-target effects have been observed through the inhibition of Human Leukocyte Elastase (HLE) [9,11,12,13], a serine protease that has been implicated in the pathogenesis of Chronic Obstructive Pulmonary Disease [14]. Thus, we hypothesized that HLE inhibitors may exhibit inhibitory activity against CtHtrA and that these chemical scaffolds may serve as a source of new lead compounds as CtHtrA inhibitors.

**Figure 2:**
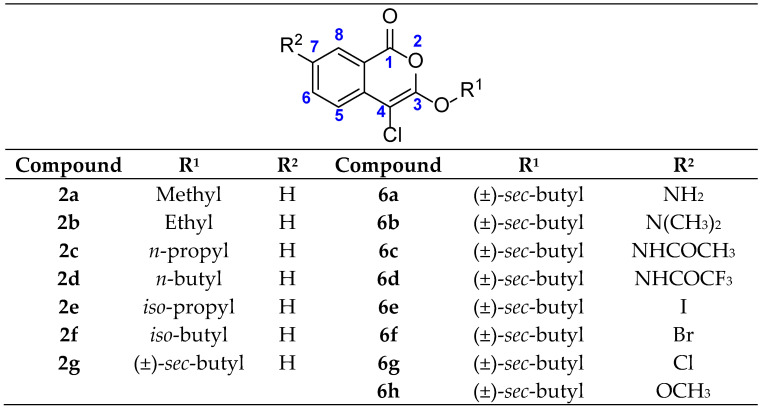
Structures of the synthesized 3-alkoxy-4-chloroisocoumarin compounds.

Isocoumarin compounds have been investigated for many years as general serine protease inhibitors, and 3,4-dichloroisocoumarin is a commercial serine protease inhibitor. In the development of HLE inhibitors, 3-alkoxy-4-chloroisocoumarins and their C-7-substituted derivatives have been identified as rapid and potent inhibitors [15,16,17,18]. As the C-3 and C-7 positions govern the selectivity and drive the inhibitory properties of 3-alkoxy-4-chloroisocoumarins against serine proteases [15,16,17,18], we focused our investigation on uncovering the structure–activity relationships (SARs) of these positions (Figure 2) with regard to their inhibitory properties against CtHtrA. We also sought to investigate the anti-chlamydial properties of the newly synthesized 4-chloroisocoumarins.

## 2. Results and Discussion

### 2.1. Design and Synthesis of 4-Chloroisocoumarin Compounds

**Scheme 1:**
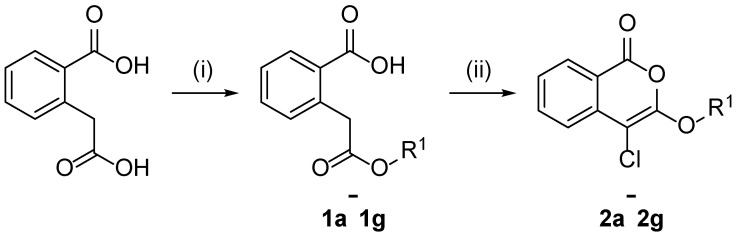
Synthesis of **2a–g**. Conditions: (**i**) R^1^OH, H_2_SO_4_, 1 h, 80 °C (or reflux for MeOH, EtOH). (**ii**) PCl_5_ (2.5 equiv.), 4 h, 40 °C.

Figure 2 shows the series of 3-alkoxy-4-chloroisocoumarin compounds synthesized in the current work. The general procedure used to prepare **2a–g** is shown in Scheme 1. Homophthalic acid was converted to the corresponding monoesters (**1a–g**) using the appropriate alcohols with sulfuric acid. Compounds **1a–g** were cyclized to the corresponding 3-alkoxy-4-chloroisocoumarin compounds (**2a–g**) using phosphorus pentachloride. The cyclization/chlorination step has been reported to take up to 16 h at 80–110 °C [19], but in our hands this reaction was complete after 4 h at 40 °C.

**Scheme 2:**
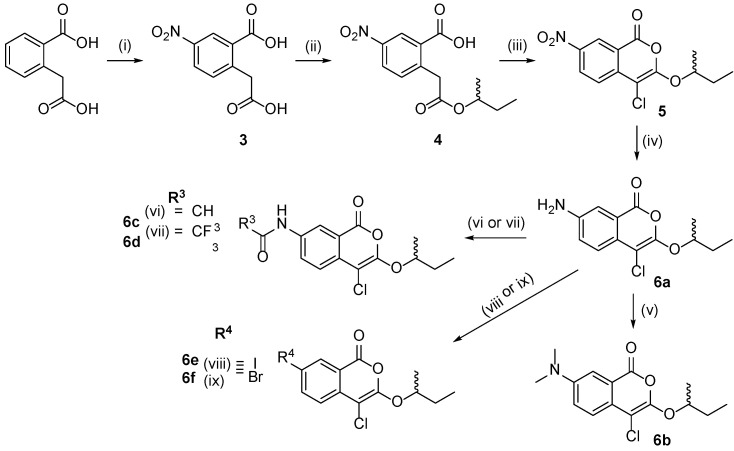
Synthesis of **6a–6f**. Conditions: (**i**) Red fuming HNO_3_, 2 h, 0–5 °C, 80%. (**ii**) (±)-*sec*-butanol, H_2_SO_4_ 18 M, 33 mol%), 7 h, 80 °C, 70%. (**iii**) PCl_5_, toluene, 4 h, reflux, 52%. (**iv**) (a) Zinc dust (10 equiv.), CH_3_COOH (10 equiv., 10% *v*/*v* in CH_2_Cl_2_), CH_2_Cl_2_, 90 min, 0–5 °C. (**v**) (a) Paraformaldehyde (10 equiv.), CH_3_COOH, 1 h, r.t. (b). NaCNBH_3_ (5 equiv.), 16 h, r.t. 35%. (**vi**) CH_3_COCl (2.5 equiv.), NaHCO_3_ (10 equiv.), CH_3_CN, 45 min, r.t. 53%. (**vii**) (CF_3_CO)_2_O (1.5 equiv.), NEt_3_ 1.75 (equiv.), CHCl_3_, 3 h, 0 °C to r.t., 76%. (**viii**) (a) HBF_4_ (1.1 equiv.), *t*-BuONO (1.1 equiv.), CH_3_CN, 10 min. 0–5 °C. (b) KI (5 equiv.), CH_3_CN/H_2_O (1:2), 1.5 h, 0–5 °C, 65%. (**ix**) (a) HBF_4_ (1.1 equiv.), *t*-BuONO (1.1 equiv.), CH_3_CN, 10 min. 0–5 °C. (b) Cetyltrimethylammonium bromide (2.5 equiv.), 3 h, r.t., 17%.

We sought to investigate the structure–activity relationship between the inhibitory properties of C-7-substituted 4-chloroisocoumarins against CtHtrA. The scope of functional groups that have been introduced at this position is limited; the current literature reports only nitrogen-based substituents [18,19,20,21]. Thus, we developed new chemistry to expand the scope of functional groups that could be introduced at this position. Based on the protease inhibition activity of **2g** (vide infra) against CtHtrA, we used this compound as a template scaffold to synthesize a range of C-7-substituted (±)-3-*sec*-butoxy-4-chloroisocoumarin compounds (Figure 2), as shown in Scheme 2.

Homophthalic acid was nitrated using red fuming nitric acid [22] and monoesterified to the corresponding *sec*-butoxy monoester. This was cyclized to give 3-*sec*-butoxy-7-nitro-4-chloroisocoumarin (**5**) and then reduced to the amine (**6a**) with zinc dust in acetic acid. Acylation to the corresponding amides and reductive double-alkylation gave the dimethylamino analogue (**6b**). Diazotization reactions were optimized to synthesize iodo (**6e**) and bromo (**6f**) analogues but did not afford the respective phenolic or chloro moieties.

**Scheme 3:**
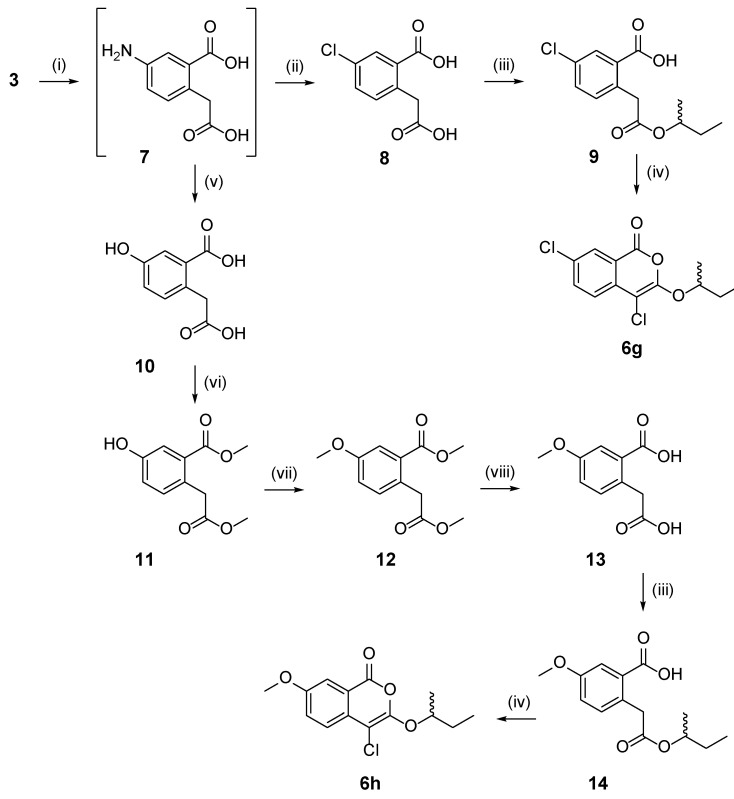
Synthesis of **6g** and **6h**. Conditions: (**i**) NH_4_CO_2_H (3.5 equiv.), Pd/C (10% *w*/*w*), methanol, 2 h, reflux. (**ii**) (a) HCl, H_2_O, NaNO_2_ (1.05 equiv.), 10 min, 0–5 °C. (b) HCl, CuCl (1.05 equiv.), 3 h, 0–5 °C, 37.1%. (**iii**) (±)-s*ec*-Butanol (4 equiv.), toluene/dioxane (5:1), H_2_SO_4_ (cat.), 6 h, 80 °C, 48% (**9**), 62%. (**14**). (**iv**) PCl_5_ (2.5 equiv.), toluene, 4 h, 40 °C, 63% (**6g**), 77% (**6h**). (**v**) (a) NaNO_2_ (1.15 equiv.), H_2_SO_4_ (4.2 M), 30 min, 0–5 °C, 79%. (b) H_2_SO_4_ (6 M), 3 h, reflux, 79%. (**vi**) 9:1 MeOH/H_2_SO_4_, 16 h, reflux, 52%. (**vii**) K_2_CO_3_ (5 equiv.), CH_3_I (2 equiv.) (CH_3_)_2_CO, 6 h, reflux, 68%. (**viii**) NaOH (3 equiv.), CH_2_Cl_2_:MeOH (9:1), 20 h, r.t, 87%.

Chloro- and methoxy-analogues (**6g** and **6h**, respectively) were synthesized using the procedure shown in Scheme 3. Compound **3** was reduced to the corresponding amine (**7**) using an adapted procedure [23] and diazotized to give 5-chlorohomophthalic acid [22] (**8**). Monoesterification with (±)-*sec*-butanol (**9**) and cyclization with phosphorus pentachloride gave the corresponding dichloroisocoumarin (**6g**). Diazotization of **7** followed by acidic thermolysis gave the corresponding phenol (**10**) [24], which was esterified to the corresponding dimethyl ester (**11**) using a Fischer esterification and then methoxylated with methyl iodide to give (**12**) [24]. The methyl ethers were hydrolyzed under basic conditions, affording (**13**) [25]. Compound **13** was monoesterified with (±)-*sec*-butanol to give (**14**) and then cyclized using the described procedure. We note that cyclization of the methoxy and chloro analogues under the reported conditions (using toluene as a solvent at reflux) did not afford the desired product; hence it may be possible that other unexplored C-7 substituents may be compatible with this chemistry.

### 2.2. Inhibitory Properties of 4-Chloroisocoumarins against CtHtrA and HLE

The inhibitory activity of compounds **2a–g** against CtHtrA was evaluated. These compounds did not exhibit substantial interference with fluorescence signals during the protease inhibition assays (see Figure S1). As these compounds are reportedly covalent inhibitors of serine proteases, a true IC_50_ is unable to be calculated. Instead, the residual activity of the protease is monitored over time in response to a range of compound concentrations, and an IC_50_ value is interpolated from a dose–response curve (see Figures S2–S4 and Tables S1 and S2). This IC_50_ value is then compared to a positive control (JO146 was used for **2a–g**, and **2g** was used for **6a–h**), giving the ‘relative potency’. This is the established method for assessing CtHtrA inhibitory potency [9,11,12]. Table 1 shows the inhibitory properties of these compounds relative to the known inhibitor JO146 (Figure 1). Extension of the R^1^ carbon chain from methyl to ethyl (**2a** vs. **2b**) gave a 1.5-fold increase in activity (relative to JO146), and potency reduced as the chain length was extended (**2b** vs. **2c** and **2d**). This effect may be due to depth restrictions within the S1 subpocket, as kinetic studies on the rates of protease inhibition in HLE were reduced by larger C-3 alkoxy substituents [17]. Chain branching (*iso*-propyl and *sec*-butyl substituents) increased the activity, resulting in a 1.4-fold increase for **2e** over **2b** and a 2.8-fold improvement for **2g** over **2c**. However, the *iso*-butyl analogue **2f** exhibited a slight reduction in activity compared to **2c**, which suggests that elongation or increases in steric bulk toward the end of the aliphatic chain are not well tolerated.

C-7 functionalized 4-chloro-3-*sec*-butoxyisocoumarins **6a**–**h** were compared against **2g** rather than JO146, as **2g** is structurally more comparable, and improvements to its potency were the aim of this investigation. Table 1 shows that all C-7 modifications resulted in reduced activity compared to **2g**. Compounds substituted with small, polar and electron-donating groups were identified as the most potent compounds in this series (**6a**–**c** and **6h**), with the exception of **6g**, which had the highest activity.

Substituent lipophilicity, as determined from their substituent hydrophobicity constants (π; see Table S3) [26], was identified as a significant factor in attenuating the CtHtrA inhibitory properties at the C-7 position. In general, the compounds bearing lipophilic substituents had little to no activity (**6d** and **6e**), while analogues with less lipophilic substituents were better CtHtrA inhibitors (**6e**–**g**, **6a** vs. **6b** and **6c** vs. **6d**).

As compounds **2a**–**g** showed effective inhibition of CtHtrA relative to JO146, attention was turned to evaluating their inhibitory properties against HLE to ascertain selectivity between the two proteases. As shown in Table 1, **2a**–**g** showed poor to modest inhibition of HLE in comparison to JO146, possibly due to their reportedly high molecular turnover, as 3-choro- and 3,4-dichloroisocoumarin have a molecular turnover of 15 and 3.1, respectively, against HLE [16]. Compound **2a** showed the poorest inhibition relative to JO146. Substitution with longer carbon chains (**2c** and **2d** vs. **2a** and **2b**) produced the most active inhibitors, potentially owing to better stabilization within the S1 subpocket after hydrolysis. Similar to their inhibitory properties against CtHtrA, **2e** and **2g** afforded the most potent inhibition of HLE in this series. This suggests comparable subpocket homology between the two proteases.

4-Chloroisocoumarins **2a–g** were found to inhibit CtHtrA preferentially over HLE. Compounds **2a** and **2b** showed a strong preference for the inhibition of CtHtrA, but this is likely due to their preference for hydrolysis by HLE. Of note, the increase in aliphatic chain length increased the selectivity toward HLE (**2a**–**d**). Isomerization of the butyl chain resulted in a slight preference for HLE (**2d** and **2f**), while **2c** and **2e** and also **2d** and **2g** showed a significant increase in preference for CtHtrA. This finding indicates that substitution at the C-1′ position of the aliphatic chain could be a driver for selectivity between the two enzymes, with **2g** showing the most promise as a CtHtrA inhibitor.

### 2.3. 4-Chloroisocoumarin Cytotoxic Effects in HEp-2 Cells and McCoy B Cells

**Figure 3:**
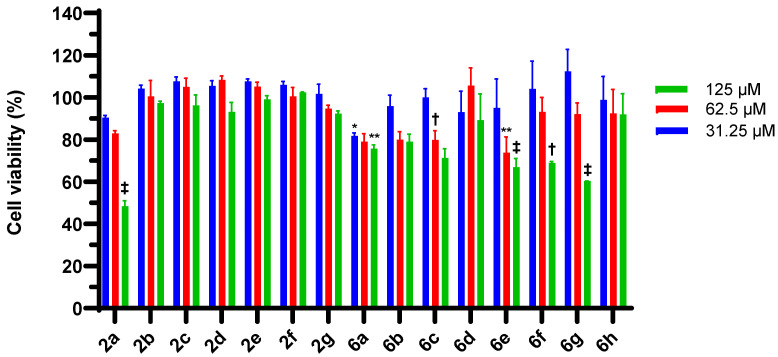
Cytotoxicity of **2a–g** and **6a–h** against HEp-2 cells (24 h, 31.25 µM, 62.5 µM and 125 µM). Values represent the average ± SEM from three independent experiments. Significance shown is relative to solvent (0.1% DMSO) controls: (*) *p* ≤ 0.05, (**) *p* ≤ 0.01, (†) *p* ≤ 0.005, (‡) *p* ≤ 0.001.

The cytotoxic effects of the 4-chloroisocoumarin compounds **2a**–**g** and **6a**–**h** (24 h) were tested against HEp-2 and McCoy B cells using the MTS cell viability assay. HEp-2 and McCoy B cells were selected, as these are routinely used for the cultivation of chlamydial species, and low cytotoxicity against host cells is required to evaluate the anti-chlamydial properties of a test compound [27]. As shown in Figure 3, it was found that compounds **2b**–**2g** had no effect on HEp-2 cell viability at concentrations up to 125 µM. The methyl-substituted compound **2a** reduced HEp-2 cell viability to 50% of DMSO-treated control cells at 125 µM but did not produce significant effects at the lower concentrations of 62.5 and 31.25 µM.

Substitution of the C-7 position in **2g** with halogens (**6e**–**6g**) increased the cytotoxicity against HEp-2 cells at 125 µM, while only **6e** retained some cytotoxicity at 62.5 µM, as shown in Figure 3. Compound **6a** reduced cell viability slightly at 125 µM, while **6b** was not found to significantly impact cytotoxicity. Acetamides **6c** and **6d** and the methoxy analogue **6h** did not significantly affect cell viability at any tested concentration. From these data, it was determined that HEp-2 cells could be used as a host cell for anti-chlamydial testing at a concentration of 62.5 µM, as this was the highest concentration that could be used without having a large impact on HEp-2 cell viability across the tested concentration range. Compounds were also tested against McCoy B cells (see Figure S5) but exhibited cytotoxicity at 31.25 and 62.5 µM, and thus this cell line was unable to be used for the cultivation of chlamydia to evaluate anti-chlamydial properties.

### 2.4. 4-Chloroisocoumarin Compound Effects against C. trachomatis

The workflow for the evaluation of chlamydial growth inhibition is described in the supplementary information (Figure S6). Briefly, HEp-2 cells were seeded in a 96-well plate and grown for 24 h; they were then infected with a fixed quantity of *C. trachomatis*. The infection was propagated for 4 h, and then a mixture of test compound and cycloheximide was added and the plate was incubated for a further 26 h (30 h post infection). The cells were fixed with methanol and then stained with fluorescent antibodies to visualize chlamydial inclusions, and the growth was measured using inclusion counts relative to vehicle controls.

**Figure 4:**
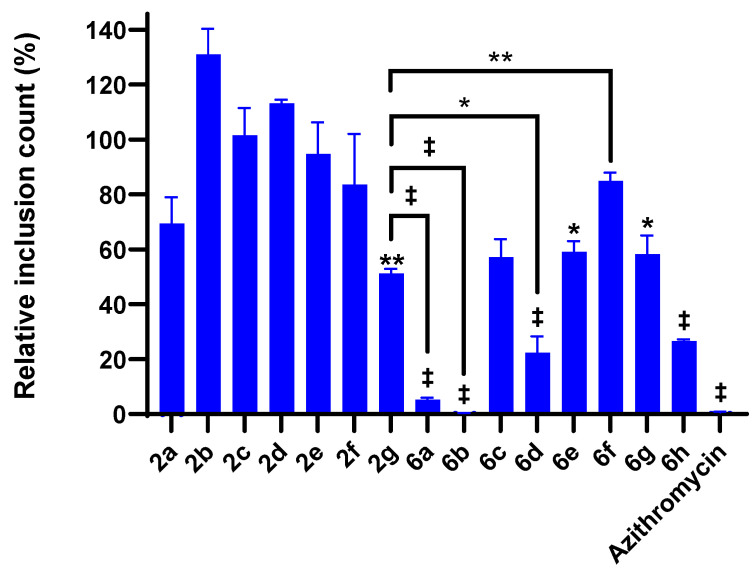
Inhibition of **2a–g** and **6a–h** against *C. trachomatis* (30 h post infection, 62.5 µM). Azithromycin positive control was used at 0.5 µg/mL. Values represent the average ± SEM from three independent experiments. Significance shown is relative to solvent (0.1% DMSO) controls: (*) *p* ≤ 0.05, (**) *p* ≤ 0.01, (‡) *p* ≤ 0.001.

The 3-alkoxy-4-chloroisocoumarins **2a–2f** did not significantly reduce the proliferation of *C. trachomatis*, with the exception of **2g**, which reduced chlamydial growth by 49% (Figure 4). C-7-substituted derivatives of **2g** were generally more active than the unsubstituted analogue **2g**. Amino (**6a**) and dimethylamino (**6b**) analogues successfully showed that ≥90% of chlamydial inclusions were affected, suggesting that small polar electron-donating groups at the C-7 position facilitate inhibition of chlamydial growth against *C. trachomatis*. Consistent with this, the methoxy substituted analogue **6h** had similar levels of activity. The acetamido (**6c**), iodo (**6e**) and chloro (**6g**) moieties did not significantly affect the efficacy of **2g** at 62.5 µM, while bromination (**6f**) abolished observable activity. Trifluoromethylacetamide (**6d**) was found to decrease chlamydial growth with comparable efficacy to the methoxy (**6h**) analogue.

Compounds with statistically significant anti-chlamydial activity were generally found to be poor inhibitors of CtHtrA (**6a**, **6b**, **6d**, **6e**, **6g**, **6h**), while compounds with poor inhibitory activity against *C. trachomatis* proliferation exhibited good activity against CtHtrA (**2a–g**). Although this illustrates that C-7-substituted 4-chloroisocoumarins can attenuate the growth of *C. trachomatis*, it is likely that their activity is not due to the inhibition of CtHtrA.

**Figure 5:**
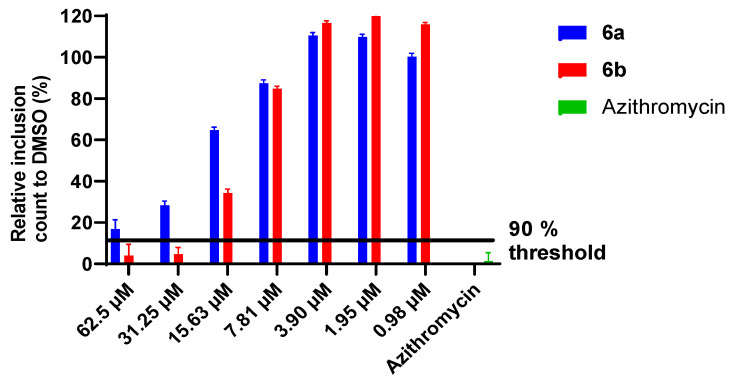
Dose–response relationships for **6a** and **6b** against *C. trachomatis* (30 h post infection). Values represent the average ± standard error of the mean from three independent experiments.

Compounds **6a** and **6b** were further investigated as they exhibited the most activity against *C. trachomatis* at 62.5 µM. In anti-chlamydial assays, the MIC (minimum inhibitory concentration) of a compound is defined as twice the MIC_TP_ (the MIC transition point), which is the concentration required to alter >90% chlamydial morphology (i.e., a reduction in size) [27]. For **6a** and **6b**, dose–response relationships for their anti-chlamydial activity were identified (Figure 5). However, **6a** did not consistently show inhibition greater than the 90% threshold at any concentration tested. Compound **6b** showed greater than 90% inhibition at concentrations ≥ 31.25 µM. These results show that while both **6a** and **6b** exhibit dose–response relationships against the inhibition of *C. trachomatis* growth, only compound **6b** has a calculable MIC of 62.5 µM, as per the reported definition [27].

## 3. Materials and Methods

### 3.1. Chemistry

Chemical reagents were purchased from Merck (Darmstadt, Germany) and used without further purification. Compounds **3** [22], **7** [23], **8** [22], **10** [24]**, 12** [24] and **13** [25] were prepared by procedures from the literature. Thin-layer chromatography was performed using Merck silica gel 60 F_254_ aluminium-backed plates. Reaction products were purified either using recrystallization or dry column vacuum chromatography on silica gel using gradient elutions as specified. NMR spectra were recorded on an Agilent 500 MHz or a Bruker 400 MHz spectrometer operating at 500 or 400 MHz for ^1^H NMR, respectively, and 125 or 100 MHz for ^13^C NMR, respectively. Spectra were referenced to the residual non-deuterated solvent signal using either CDCl_3_ (^1^H δ 7.26, ^13^C 77.00) or DMSO-*d*_6_ (^1^H δ 2.50, ^13^C δ 39.52). Multiplicity was assigned as singlet (s), doublet (d), triplet (t), quartet (q), quintet (quin), sextet (sxt) or multiplet (m). High-resolution mass spectra (HRMS) were recorded on an Agilent technologies 6510 Q-TOF MS. Compound purity was confirmed to be >95% prior to biological assays using quantitative NMR spectroscopy (see supplementary information) [28,29].

#### 3.1.1. General Procedure for the Synthesis of Unsubstituted Homophthalate Monoesters (**1a–1g**)

Homophthalic acid (1.5 g, 8.3 mmol) was dissolved in the corresponding anhydrous alcohol (20 equiv.), to which sulfuric acid (98%, 1 mL) was added. The reaction mixture was heated under reflux for methyl, ethyl and isopropyl alcohols, and at 80 °C for other alcohols, until the starting material was no longer present by TLC analysis (ethyl acetate mobile phase, typically 1–1.5 h, except the methyl ester, which took 30 min). Work-up for methyl and ethyl analogues followed work-up procedure A. All other analogues followed work-up procedure B.

Work-up A: The reaction was diluted with ethyl acetate (30 mL) and washed with saturated sodium bicarbonate (3 × 50 mL). The organic phase was removed, and the aqueous phase was acidified to pH 2–3 with aqueous hydrochloric acid (1 M) and then washed with ethyl acetate (3 × 50 mL). The combined extracts were washed with brine (100 mL), dried using anhydrous magnesium sulfate and then concentrated with reduced pressure to afford solids that were crystallized from 10% ethyl acetate in hexane.

Work-up B: The reaction was diluted with chloroform (50 mL) and washed with water (2 × 100 mL) and then brine (50 mL) and dried using anhydrous magnesium sulfate. The solvent was removed with reduced pressure and dissolved in a minimal amount of ethyl acetate (typically 2–3 mL) at 60 °C and then slowly diluted with hexanes until the solution became slightly cloudy. The mixture was cooled to room temperature over 30 min and then further with an ice bath for one hour. The resulting white needles were filtered and washed with hexanes (2 × 20 mL) and dried with reduced pressure for 16 h. ^1^H, ^13^C NMR spectra and melting points for compounds **1a–e** were consistent with those previously reported [19,30].

2-(2-*iso*-butoxy-2-oxo-ethyl)benzoic acid (**1f**). White solid (0.800 g, 49%) ^1^H NMR: (400 MHz, CDCl_3_) δ 11.33 (br s, 1H, COOH), 8.13 (dd, J = 1.2, 8.0 Hz, 1H, H-6), 7.54 (td, J = 1.2, 7.6 Hz, 1H, H-5), 7.40 (td, J = 1.2, 7.6 Hz, 1H, H-4), 7.29 (d, J = 7.6 Hz, 1H, H-3), 4.08 (s, 2H, CH_2_CO_2_), 3.88 (d, 2H, OCH_2_), 1.91 (sxt, J = 6.8 Hz, 1H, CH), 0.90 (d, J = 6.8 Hz, 6H, 2 × CH_3_). ^13^C NMR: (100 MHz, CDCl_3_) δ 172.2, 171.5, 137.0, 133.2, 132.4, 131.9, 128.6, 127.5, 70.9, 40.7, 27.7, 19.0. HRMS (ESI/Q-TOF) *m*/*z*: [M + Na]^+^ Calcd for C_13_H_16_O_4_Na 259.0946; Found 259.0951. m.p. 120–122 °C.
(±)-2-(2-*sec*-butoxy-2-oxo-ethyl)benzoic acid (**1g**). White solid (1.22 g, 62%), ^1^H NMR: (400 MHz, CDCl_3_) δ 8.12 (dd, J = 7.6, 1.2 Hz, 1H, H-6), 7.53, (td, J = 1.2 Hz, 7.6 Hz, 1H, H-5), 7.40 (td, J = 1.2, 7.6 Hz, 1H, H-4), 7.28 (d, J = 7.6 Hz, 1H, H-3), 4.87 (sxt, J = 6.4 Hz, 1H, C-1′), 4.03 (d, J = 2.0 Hz, 2H, CH_2_CO_2_), 1.66–1.47, m, 2H, CH_2_), 1.21 (d, J = 6.4 Hz, 3H, CH_3_), 0.88 (t, J = 7.2 Hz, 3H, CH_3_). ^13^C NMR: (100 MHz, CDCl_3_) δ 172.5, 171.2, 137.1, 133.2, 132.4, 131.8, 128.7, 127.4, 72.8, 41.1, 28.7, 19.3, 9.6. HRMS (ESI/Q-TOF) *m*/*z*: [M + Na]^+^ Calcd for C_13_H_16_O_4_Na 259.0946; Found 259.0942. m.p. 101–103 °C.

#### 3.1.2. General Procedure for the Synthesis of 3-alkoxy-4-chloroisocoumarins (**2a–2g**) (General Procedure A)

This procedure was adapted from the literature [19]. Homophthalate monoester (up to 4 g) was dissolved in anhydrous toluene (120 mL/g), and the flask was backfilled three times with argon gas. Phosphorus pentachloride (3.0 equiv.) was added in a single portion with stirring, and the flask was evacuated for one minute and then backfilled with argon gas and stirred at 40 °C for four hours. The reaction was cooled on an ice bath, and water (50 mL) was added and stirred while cold for five minutes. The toluene phase was separated and washed sequentially with water (50 mL), saturated aqueous sodium bicarbonate (50 mL) and brine (50 mL) and then dried using magnesium sulfate and concentrated with reduced pressure to afford a yellow or orange oil that was purified on silica gel (0–20% dichloromethane in hexanes) and then recrystallized by heating to 40 °C in hexane and then cooling to −15 °C (approximately 1 mL of hexane per 100 mg of material).

3-Methoxy-4-chloroisocoumarin (**2a**). Pale-yellow needles (0.272 g, 20%). ^1^H NMR (500 MHz, CDCl_3_) δ 8.20 (d, J = 7.5 Hz, H-8), 7.77–7.72 (m, 2H, H-6 and H-5), 7.39 (dt, J = 8.5, 1.0 Hz, 1H, H-7), 4.46 (q, J = 7.0 Hz, 2H, CH_2_), 1.47 (t, J = 7.0 Hz, 3H, CH_3_). ^13^C NMR (125 MHz, CDCl_3_) δ 159.7, 153.1, 137.9, 135.6, 130.1, 126.2, 122.23 117.3, 91.3, 66.8, 14.9. HRMS (ESI/Q-TOF) *m*/*z*: [M + Na]^+^ Calcd for C_11_H_10_ClO_3_Na 247.0138; Found 247.0127. ^1^H, ^13^C NMR and melting point was consistent with previous reported data [31].
3-Ethoxy-4-chloroisocoumarin (**2b**). Pale-yellow needles (0.250 g, 23%). ^1^H NMR (500 MHz, CDCl_3_) δ 8.20 (d, J = 7.5 Hz, H-8), 7.77–7.72 (m, 2H, H-6 and H-5), 7.39 (dt, J = 8.5, 1.0 Hz, 1H, H-7), 4.46 (q, J = 7.0 Hz, 2H, CH_2_), 1.47 (t, J = 7.0 Hz, 3H, CH_3_). ^13^C NMR (125 MHz, CDCl_3_) δ 159.7, 153.1, 137.9, 135.6, 130.1, 126.2, 122.23 117.3, 91.3, 66.8, 14.9. HRMS (ESI/Q-TOF) *m*/*z*: [M + Na]^+^ Calcd for C_11_H_10_ClO_3_Na 247.0138; Found 247.0127. m.p. 94–96 °C. Melting point was consistent with the reported value [31].
3-Propoxy-4-chloroisocoumarin (**2c**). ^1^H, ^13^C NMR and melting point were consistent with those previously reported [19].
3-Butoxy-4-chloroisocoumarin (**2d**). ^1^H, ^13^C NMR and melting point were consistent with those previously reported [19].
3-*iso*-Propoxy-4-chloroisocoumarin (**2e**). Pale yellow solid (0.235 g, 34%) ^1^H NMR: (500 MHz, CDCl_3_) δ 8.21 (d, J = 8.0 Hz, 1H, H-8), 7.78–7.71 (m, 2H, H-6 and H-5), 7.40 (dt, J = 7.0, 1.5 Hz, 1H, H-7), 5.06 (sep, J = 6.0 Hz, 1H, CH), (d, J = 6.0 Hz, 6H, 2 × CH_3_). ^13^C NMR: (125 MHz, CDCl_3_) δ 160.0, 152.7, 137.8, 135.6, 130.1, 126.3, 122.5, 117.6, 93.0, 75.6, 22.3. HRMS (ESI/Q-TOF) *m*/*z*: [M + Na]^+^ Calcd for C_12_H_11_ClO_3_Na 261.0289; Found 261.0284. m.p. 58–60 °C.
3-*iso*-Butoxy-4-chloroisocoumarin (**2f**). Yellow solid (0.255 g, 34%) ^1^H NMR: (500 MHz, CDCl_3_) δ 8.20 (dd, J = 7.5, 1.5 Hz, 1H, H-8), 7.77–7.70 (m, 2H, H-6 and H-5), 7.39 (dt, J = 7.0, 1.5 Hz, 1H, H-7), 4.15 (d, J = 6.5 Hz, 2H, CH_2_), (sep, J = 7.0 Hz, 1H, CH), 1.05 (d, J = 7.0 Hz, 6H, 2 × CH_3_).^13^C NMR: (125 MHz, CDCl_3_) δ 159.7, 153.3, 138.0, 135.6, 130.1, 126.1, 122.2, 117.3, 91.0, 76.6, 28.4, 18.8. HRMS (ESI/Q-TOF) *m*/*z*: [M + Na]^+^ Calcd for C_13_H_13_ClO_3_Na 275.0451; Found 275.0444. m.p. 48–51 °C. Melting point was consistent with the reported value [16].
(±)-3-*sec*-Butoxy-4-chloroisocoumarin (**2g**). Yellow solid (0.215 g, 28%) ^1^H NMR: (500 MHz, CDCl_3_) δ 8.21 (br d, J = 7.5 Hz, 1H, H-8), 7.77–7.71 (m, 2H, H-6 and H-5), 7.40 (dt, J = 7.5, 1.5 Hz, 1H, H-7), 4.87 (sxt, J = 6.0 Hz, 1H, CH), 1.86–1.66 (m, 2H, CH_2_), 1.40 (d, J = 6.5 Hz, 3H, OCHC***H_3_***), 1.03 (t, J = 7.5 Hz, 3H, CH_2_C***H_3_***). ^13^C NMR: (125 MHz, CDCl_3_) δ 160.0, 152.9, 137.9, 135.6, 130.1, 126.2, 122.4, 117.6, 92.6, 80.2, 29.2, 19.8, 9.5. HRMS (ESI/Q-TOF) *m*/*z*: [M + Na]^+^ Calcd for C_13_H_13_O_3_ClNa 275.0451; Found 275.0439. m.p. 57–59 °C.

#### 3.1.3. General Procedure for the Synthesis of 5-Substituted Homophthalate Monoesters (**9** and **13**) (General Procedure B)

The respective homophthalic acid derivative was dissolved in a mixture of anhydrous toluene and 1,4-dioxane 5:1 (5 mL/mmol) to which (±)-*sec*-butanol (4 equiv.) was added. Sulfuric acid (18 M, 20 drops/g) was added, and the mixture was heated to 90 °C for six hours. The mixture was cooled to room temperature and diluted with chloroform (30 mL/g), washed sequentially with water (3 × 75 mL) and brine (75 mL) and then dried using anhydrous magnesium sulfate. The organic phase was concentrated with reduced pressure and the oil was recrystallized with a mixture of ethyl acetate in hexane.

(±)-5-Chloro-2-(2-*sec*-butoxy-2-oxo-ethyl)benzoic acid (**9**). White solid (0.300 g, 48%). ^1^H NMR: (400 MHz, CDCl_3_) δ 10.66 (br s, 1H, COOH), 8.09 (d, J = 2.4 Hz, 1H, H-6), 7.50 (dd, J = 2.4, 8.0 Hz, 1H, H-4), 7.22 (d, J = 7.6 Hz, 1H, H-3), 4.86 (sxt, J = 6.4 Hz, 1H, CH), 3.99 (d, J = 2.0 Hz, 2H, CH_2_CO_2_), 1.65–1.47 (m, 2H, CH_2_), 1.20 (d, J = 6.4 Hz, 3H, OCHC***H_3_***), 0.88 (t, J = 7.6 Hz, 3H, CH_2_C***H_3_***). ^13^C NMR: (100 MHz, CDCl_3_) δ 171.3, 170.7, 135.5, 133.7, 133.4, 133.2, 131.7, 130.1, 73.1, 40.4, 28.7, 19.3, 9.6. HRMS (ESI/Q-TOF) *m*/*z*: [M + Na]^+^ Calcd for C_13_H_15_O_4_Na 293.0557; Found 293.0548. m.p. 100–103 °C.
(±)-5-Methoxy-2-(2-*sec*-butoxy-2-oxo-ethyl)benzoic acid (**13**). Brown solid (1.53 g, 62%) ^1^H NMR: (400 MHz, CDCl_3_) δ 7.64 (d, *J* = 2.8 Hz, 1H, H-6), 7.18 (d, *J* = 8.4 Hz, 1H, H-3), 7.07 (dd, *J* = 3.2 Hz, 8.4 Hz, 1H, H-4), 4.86 (sxt, *J* = 6.4 Hz, 1H, CH), 3.95 (d, *J* = 2.0 Hz, 2H, CH_2_CO_2_), 3.85 (s, 3H, OCH_3_), 1.65-1.43 (m, 2H, CH_2_), 1.20 (d, *J* = 6.0 Hz, 3H, OCHC***H_3_***), 0.88 (d, *J* = 7.6 Hz, 3H, CH_2_C***H_3_***). ^13^C NMR: (100 MHz, CDCl_3_) δ 172.4, 171.6, 158.5, 133.4, 129.5, 129.2, 119.3, 116.5, 72.7, 55.5, 40.2, 28.7, 19.3, 9.6. HRMS (ESI/Q-TOF) *m*/*z*: [M + Na]^+^ Calcd for C_13_H_18_O_5_Na 289.1052; Found 289.1048. m.p. 115–118 °C.

#### 3.1.4. Synthesis of (±)-5-Nitro-2-(2-sec-butoxy-2-oxo-ethyl)benzoic acid (**4**)

Compound **3** (6.90 g, 30.6 mmol) was suspended in *sec*-butanol (60.0 mL, 48.5 g, 21.4 equiv.) to which concentrated sulfuric acid (18 M, 2.0 mL) was added dropwise with vigorous stirring at room temperature. The solution was heated to 80 °C and stirred for eight hours. The solution was cooled to room temperature and concentrated to approximately 20–25 mL with reduced pressure at 55 °C. Chloroform (50 mL) was added, and the solution was washed sequentially with water (2 × 75 mL) and brine (75 mL) and then dried with magnesium sulfate and concentrated to give a viscous brown oil. Ethyl acetate (5 mL) was added, and the solution was heated to 70 °C and slowly diluted with hot hexanes (approximately 45 mL) until trace precipitation was observed. The solution was cooled slowly to room temperature and stored at −20 °C for 16 h. The solid was collected by filtration and washed with hexanes (2 × 10 mL) to afford the title compound.

(±)-5-Nitro-2-(2-*sec*-butoxy-2-oxo-ethyl)benzoic *acid* (**4**) White solid (6.50 g, 76%) ^1^H NMR: (400 MHz, CDCl_3_) δ 8.95 (s, J = 2.4 Hz, 1H, H-6), 8.38 (dd, J = 2.4 Hz, 8.4 Hz, 1H, H-4), 7.50 (d, J = 8.4 Hz, 1H, H-3), 4.88 (sxt, J = 6.2 Hz, 1H, CH), 4.16 (d, J = 2.8 Hz, 2H, H-2′), 1.66–1.50 (m, 2H, C***H*_2_**CH_3_), 1.22 (d, J = 6.2 Hz, 3H, OCHC***H_3_***), 0.88 (t, J = 7.4 Hz, 3H, CH_2_C***H_3_***). ^13^C NMR: (100 MHz, CDCl_3_) δ 170.3, 169.8, 147.1, 143.9, 133.7, 130.02, 127.4, 126.8, 73.6, 40.9, 28.7, 19.3, 9.6. HRMS (ESI/Q-TOF) *m*/*z*: [M + Na]^+^ Calcd for C_13_H_15_NO_6_Na 304.0797; Found 304.0790. m.p. 112–115 °C.
(±)-3-*sec*-Butoxy-7-nitro-4-chloroisocoumarin (**5**). Synthesized using general procedure A with minor modifications. The reaction was performed at reflux and the product was triturated with methanol to afford yellow needles (2.49 g, 59%). ^1^H NMR: (400 MHz, CDCl_3_) δ 9.02 (d, *J* = 2.0 Hz, 1H, H-8), 8.51 (dd, *J* = 9.2, 2.4 Hz, 1H, H-6), 7.82 (d, *J* = 8.8 Hz, 1H, H-5), 5.02 (m, 1H, CH), 1.90–1.70 (m, 2H, CH_2_), 1.45 (d, *J* = 6.2 Hz, 3H, OCHC***H_3_***), 1.03 (t, *J* = 7.4 Hz, 3H, CH_2_C***H_3_***). ^13^C NMR: (100 MHz, CDCl_3_) δ 157.8, 155.7, 145.0, 143.2, 129.6, 126.3, 123.5, 116.8, 90.8, 81.2, 29.2, 20.0, 9.4. HRMS (ESI/Q-TOF) *m*/*z*: [M + Na]^+^ Calcd for C_13_H_12_NO_5_ClNa 320.0302; Found 320.0292. m.p. 108–111 °C.

#### 3.1.5. Synthesis of (±)-7-Amino-3-sec-butoxy-4-chloroisocoumarin (**6a**)

Compound **5** (2.0 g, 6.71 mmol) was dissolved in anhydrous dichloromethane (80 mL) to which activated zinc dust*^a^* (4.39 g, 10 equiv.) was added and stirred vigorously at 0 °C for ten minutes. Glacial acetic acid (4.03 g, 3.84 mL, 10 equiv.) was dissolved in anhydrous chloroform (40 mL) and added dropwise over 30 min while maintaining a temperature of below 5 °C, and then it was stirred for a further hour. Excess zinc dust was removed by filtration, and saturated sodium bicarbonate (100 mL) was added, and the mixture was stirred vigorously for ten minutes at room temperature. The precipitated salts were removed by filtration, and the organic phase was removed, washed with water (2 × 100 mL) and brine (100 mL), and then dried using anhydrous magnesium sulfate. The solvent was removed with reduced pressure giving a bright yellow solid. An analytical sample was recrystallized from petroleum ether (80–110 °C) for use in biological assays.

(±)-7-Amino-4-chloro-3-*sec*-butoxyisocoumarin (**6a**). Yellow solid (1.70 g, 95%) ^1^H NMR: (400 MHz, CDCl_3_) δ 7.53 (d, *J* = 8.8 Hz, 1H, H-5), 7.45 (d, *J* = 2.4 Hz, 1H, H-8), 7.10 (dd, *J* = 8.8, 2.4 Hz, 1H, H-6), 4.72 (sxt, *J* = 6.0 Hz, 1H, CH), 3.95 (br s, 2H, NH_2_), 1.84–1.63 (m, 2H, CH_2_), 1.35 (d, *J* = 6.0 Hz, 3H, OCHC***H_3_***), 1.01 (t, *J* = 7.6 Hz, 3H, CH_2_C***H_3_***). ^13^C NMR: (100 MHz, CDCl_3_) δ 160.4, 150.5, 145.4, 128.8, 123.9, 123.6, 119.2, 113.1, 94.2, 80.4, 29.2, 19.6, 9.5. HRMS (ESI/Q-TOF) *m*/*z*: [M + Na]^+^ Calcd for C_13_H_14_ClNO_3_Na 290.0560; Found 290.0566. m.p. 86–88 °C.

Zinc dust was activated by stirring 10% wt powdered zinc in 5% aqueous hydrochloric acid for five minutes and then filtering and washing sequentially with water (2 × 50 mL), methanol (2 × 50 mL) and diethyl ether (2 × 25 mL). The dust was then dried at 50 °C with reduced pressure for 30 min and stored under vacuum.

#### 3.1.6. Synthesis of (±)-7-(dimethylamino)-3-sec-butoxy-4-chloroisocoumarin (**6b**)

Compound **6a** (0.400 g, 1.50 mmol) was added to glacial acetic acid (12 mL) in a sealed vial, and the mixture was stirred vigorously until all material dissolved. Paraformaldehyde (0.449 g, 10 equiv.) was added and stirred at room temperature with the vial sealed for one hour. Sodium cyanoborohydride (0.470 g, 5 equiv.) was added and stirred overnight. The reaction mixture was neutralized by slow addition to saturated aqueous sodium carbonate (100 mL) and then extracted with chloroform (3 × 50 mL). The combined extracts were washed with water (2 × 100 mL) and brine (100 mL) and dried using anhydrous potassium carbonate. The solvent was removed with reduced pressure and purified using gradient silica gel column chromatography with the crude mixture loaded using 10% dichloromethane in hexane and eluted with 25–50% dichloromethane in hexane in 5% increments to afford a bright yellow oil (0.154 g, 35%).

(±)-7-(Dimethylamino)-3-sec-butoxy-4-chloroisocoumarin (**6b**). ^1^H NMR: (400 MHz, CDCl_3_) δ 7.58 (d, *J* = 8.8 Hz, 1H, H-8), 7.39 (d, *J* = 2.8 Hz, 1H, H-5), 7.18 (dd, *J* = 2.8, 9.2 Hz, 1H, H-6), 4.72 (sxt, 6.0 Hz, 1H, CH), 3.03 (s, 6H, N(CH_3_)**_2_**), 1.85–1.63 (m, 2H, CH_2_), 1.36 (d, *J* = 6.4 Hz, 3H, OCHC***H_3_***), 1.02 (t, *J* = 7.6 Hz, 3H, CH_2_C***H_3_***). ^13^C NMR: (100 MHz, CDCl_3_) δ 160.9, 150.1, 149.1, 126.6, 123.6, 120.8, 119.1, 110.3, 94.5, 80.3, 40.5, 29.2, 19.6, 9.5. HRMS (ESI/Q-TOF) *m*/*z*: [M + Na]^+^ Calcd for C_15_H_18_ClNO_3_Na 318.0867; Found 318.0870.

#### 3.1.7. Synthesis of (±)-N-(4-Chloro-1-oxo-3-sec-butoxy-isochromen-7-yl)acetamide (**6c**)

Compound **6a** (0.250 g, 0.935 mmol) was dissolved in acetonitrile (15 mL), to which anhydrous sodium bicarbonate (0.785 g, 10 equiv.) was added and stirred vigorously at 0 °C for five minutes. Acetyl chloride (0.166 µL, 183 mg, 2.5 equiv.) was added in a single portion and stirred for 45 min while during which the mixture warmed to room temperature. The solution was diluted with ethyl acetate (40 mL) and washed sequentially with water (2 × 50 mL) and brine (50 mL) and then dried using anhydrous magnesium sulfate and concentrated with reduced pressure to afford a light yellow waxy solid. The solid was recrystallized from 5 mL of 3:7 ethyl acetate in hexane and washed with hexane (2 × 10 mL), affording the title compound as small pale-yellow needles (0.160 g, 55%).

(±)-N-(4-Chloro-1-oxo-3-sec-butoxy-isochromen-7-yl)acetamide (**6c**). ^1^H NMR: (400 MHz, CDCl_3_) δ 8.21 (dd, J = 2.1 Hz, 8.8 Hz, 1H, H-6), 8.11 (d, J = 2.2 Hz, 1H, H-8), 7.69 (d, J = 8.8 Hz, 1H, H-5), 7.61 (br s, 1H, NH), 4.85–4.78 (m, 1H, CH), 2.23 (s, 3H, NHCOCH_3_), 1.85-1.67 (m, 2H, CH_2_), 1.38 (d, J = 6.2 Hz, 3H, OCHC***H_3_***), 1.02 (t, J = 7.4, 3H, CH_2_C***H_3_***). ^13^C NMR: (100 MHz, CDCl_3_) δ 168.9, 160.0, 152.1, 136.9, 133.8, 128.0, 123.5, 119.4, 117.9, 93.2, 80.6, 29.2, 24.4, 19.8, 9.5. HRMS (ESI/Q-TOF) *m*/*z*: [M + Na]^+^ Calcd for C_15_H_16_ClNO_4_Na 332.0660; Found 332.0660,. m.p. 161 °C (decomposed with gas evolution).

#### 3.1.8. (±)-N-(4-chloro-1-oxo-3-sec-butoxy-isochromen-7-yl)-2,2,2-trifluoroacetamide (**6d**)

Compound **6a** (0.267 g, 1.0 mmol) was dissolved in anhydrous chloroform (10 mL) and cooled to 0–5 °C. Trifluoroacetic anhydride (209 µL, 313 mg, 1.5 equiv.) was added dropwise as a solution in chloroform (2.0 mL). Triethylamine (219 µL, 170 mg, 1.75 equiv.) was added dropwise as a solution in chloroform (2.0 mL) and stirred for 10 min and then warmed to room temperature and stirred for three hours. The solution was diluted with chloroform (15 mL) and washed sequentially with saturated aqueous sodium bicarbonate (30 mL), water (30 mL) and brine (30 mL) and then dried using anhydrous magnesium sulfate. The solvent was removed with reduced pressure and then the crude solid was dissolved in chloroform at 60 °C (4.0 mL), and n-hexane (4.0 mL) was added slowly with mixing. The solution was cooled to room temperature over 30 min, and yellow needles were observed. The solution was stood at −20 °C overnight and the crystals were filtered and washed with a cold solution of 1:9 chloroform in hexanes (3 × 3 mL), affording a bright yellow solid (0.278 g, 76%). ^1^H NMR: (400 MHz, CDCl_3_) δ 8.76 (br s, 1H, NH), 8.40 (d, *J* = 2.0 Hz, 1H, H-8), 8.34 (dd, *J* = 2.0, 8.8 Hz, 1H, H-6), 7.78 (d, *J* = 8.8 Hz, 1H, H-5), 4.85 (sxt, *J* = 6.0 Hz, 1H, CH), 1.89–1.68 (m, 2H, CH_2_), 1.42 (d, *J* = 6.0 Hz, 3H, OCHC***H_3_***), 1.04 (t, *J* = 7.6 Hz, 3H, CH_2_C***H_3_***). ^13^C NMR: (100 MHz, CDCl_3_) δ 159.9, 155.2 (q, *J* = 38 Hz, F_3_C**C**O), 152.9, 135.8, 134.2, 128.2, 123.9, 120.8, 117.8, 115.7 (q, *J* = 287 Hz, F_3_**C**CO), 92.5, 81.0, 29.3, 19.9, 9.5. HRMS (ESI/Q-TOF) *m*/*z*: [M + Na]^+^ Calcd for C_15_H_13_ClF_3_NO_4_Na 386.0383; Found 386.0383. m.p. 189–190 °C.

#### 3.1.9. General Procedure for the Synthesis of **6e** and **6f**

Compound **6a** (up to 1.50 g, 5.60 mmol) was dissolved in anhydrous acetonitrile (10 mL/mmol) and cooled to 0–5 °C. The flask was purged under nitrogen gas for 10 min and sealed with a rubber septum; then, tetrafluoroboric acid (1.1 equiv.) was added and stirred for five minutes. *tert-*Butylnitrite (1.1 equiv.) in acetonitrile (1 mL/mmol) was added slowly and stirred for a further five minutes at 0–5 °C. Potassium iodide (5 equiv.) in 1:2 acetonitrile in water (1 mL per 1.5 mmol KI) was added over two minutes (compound **6e**), or cetyltrimethylammonium bromide (2.5 equiv.) was added as a single portion (compound **6f**). Bubbles were observed on addition, and the solution was stirred at 0–5 °C (**6e**: 1.5 h, **6f**:3 h). Work-up conditions are specified for each compound below.

(±)-3-*sec*-Butoxy-4-chloro-7-iodoisocoumarin (**6e**). The mixture was poured into an aqueous solution (100 mL) of 5% sodium bicarbonate containing sodium thiosulfate (7.5 g). The product was extracted into hexane (75 mL) and then washed with water (50 mL) and brine (50 mL) and then dried using anhydrous magnesium sulfate. The solvent was loaded onto a silica gel column and purified with a gradient of 0–20% dichloromethane in hexanes, affording a yellow solid (1.38 g, 65%).^1^H NMR: (400 MHz, CDCl_3_) δ 8.49 (d, *J* = 1.6 Hz, 1H, H-8), 7.99 (dd, *J* = 2.0, 8.4 Hz, 1H, H-6), 7.43 (d, *J* = 8.4 Hz, 1H, H-5), 4.87 (sxt, 6.0 Hz, 1H, CH), 1.86–1.59 (m, 2H, CH_2_), 1.39 (d, *J* = 6.0 Hz, 3H, CHC***H_3_***), 1.02 (t, *J* = 7.6 Hz, 3H, CH_2_C***H_3_***)**.** ^13^C NMR: (100 MHz, CDCl_3_) δ 158.4, 153.2, 144.1, 138.4, 137.3, 124.1, 118.9, 91.9, 89.6, 80.5, 29.2, 19.9, 9.5. HRMS (ESI/Q-TOF) *m*/*z*: [M + Na]^+^ Calcd for C_13_H_12_ClIO_3_Na 400.9417; Found 400.9408. m.p. 69–72 °C
(±)-7-Bromo-3-sec-butoxy-4-chloro-isocoumarin (**6f**). The solvent was removed with reduced pressure at room temperature, and the crude solid was sonicated in hexanes for 15 min. The slurry was loaded onto a silica gel column and purified with a gradient of 0–20% dichloromethane in hexanes, affording a yellow solid (0.170 g, 17%). ^1^H NMR: (400 MHz, CDCl_3_) δ 8.32 (d, *J* = 2.0 Hz, 1H, H-8), 7.82 (dd, *J* = 2.0, 8.4 Hz, 1H, H-6), 7.58 (d, *J* = 8.8 Hz, 1H, H-5), 4.87 (sxt, 6.0 Hz, 1H, CH), 1.87–1.66 (m, 2H, CH_2_), 1.39 (t, *J* = 6.4 Hz, 3H, CH_2_C***H_3_***), 1.01 (d, *J* = 6.4 Hz, 3H, CHC***H_3_***). ^13^C NMR: δ 159.6, 153.1, 138.6, 136.9, 132.3, 124.2, 119.4, 118.8, 92.0, 80.6, 29.2, 19.8, 9.5. HRMS (ESI/Q-TOF) *m*/*z*: [M + Na]^+^ Calcd for C_13_H_12_BrClO_3_Na 352.9556; Found 352.9542. m.p. 78–80 °C.

#### 3.1.10. Synthesis of Compounds **6g** and **6h**

(±)-3-*sec*-Butoxy-4,7-dichloro-isocoumarin (**6g**). Synthesized using general procedure A to afford a yellow solid (0.328 g, 63%) ^1^H NMR: (400 MHz, CDCl_3_) δ 8.16 (d, *J* = 1.6 Hz, 1H, H-8), 7.67–7.64 (m, 2H, H-5 and H-6) 4.87 (sxt, *J* = 6.4 Hz, 1H, CH), 1.87–1.66 (m, 2H, CH_2_), 1.40 (d, *J* = 6.0 Hz, 3H, OCHC***H*_3_**), 1.02 (t, *J* = 7.6 Hz, 3H, CH_2_C***H_3_***). ^13^C NMR: (100 MHz, CDCl_3_) δ 158.8, 153.1, 136.5, 135.8, 132.0, 129.3, 124.1, 118.6, 92.0, 80.6, 29.2, 19.8, 9.5. HRMS (ESI/Q-TOF) *m*/*z*: [M + Na]^+^ Calcd for C_13_H_12_Cl_2_O_3_H^+^ 309.0061; Found 309.0051. m.p. 86–88 °C.
(±)-7-Methoxy-3-*sec*-butoxy-4-chloroisocoumarin (**6h**). Synthesized using general procedure A to afford a yellow solid (0.410 g, 77%) ^1^H NMR: (400 MHz, CDCl_3_) δ 7.62 (d, *J* = 8.8 Hz, 1H, H-5), 7.59 (d, *J* = 2.8 Hz, 1H, H-8), 7.32 (dd, *J* = 8.8, 2.8 Hz, 1H, H-6), 4.78 (sxt, *J* = 6.0 Hz, 1H, CH), 3.88 (s, 3H, OCH_3_), 1.85-1.64 (m, 2H, CH_2_), 1.37 (d, *J* = 6.0 Hz, 3H, OCHC***H_3_***), 1.01 (t, *J* = 7.6 Hz, 3H, CH_2_C***H_3_***). ^13^C NMR: (100 MHz, CDCl_3_) δ 160.0, 158.2, 151.4, 131.4, 125.1, 124.1, 118.7, 110.4, 93.4, 80.4, 55.7, 29.2, 19.7, 9.4. HRMS (ESI/Q-TOF) *m*/*z*: [M + Na]^+^ Calcd for C_14_H_15_O_4_ClNa 305.0549; Found 305.0557. m.p. 55–56 °C.

### 3.2. Biological Studies

#### 3.2.1. Recombinant Expression and Purification of CtHtrA

CtHtrA was expressed using an procedure adapted from the literature [32]. Recombinant *E. coli* BL21(DE3) pET22b was grown in LB media containing 100 ug/mL ampicillin to an OD_600_ of 0.5-0.6 in 5 × 400 mL batches at 37 °C. Isopropyl β-D-1-thioglactopyranoside (1 M, 400 µL, 1 mM final concentration) was added to each flask and incubated at 30 °C with shaking at 150 rpm for an additional 2.5 h. The culture was centrifuged at 4000× *g* for 15 min at 4 °C, and the cell pellet was harvested and resuspended in lysis buffer (50 mM phosphate, 10 mM imidazole, 500 mM NaCl, 0.1% Triton X-100, 10% glycerol). Lysozyme was added to the suspended cells and was sonicated in 15 cycles of 15 s, with one minute between cycles while maintaining 4 °C, and then centrifuged at 8000× *g* for 20 min to remove any debris, retaining the supernatant for protein purification.

TALON^®^ metal affinity resin (Takara cat no. 635652, 5 mL) was added to each lysate tube and washed sequentially with lysis buffer (2 × 45 mL) and stringency buffer (4 × 45 mL) and then eluted with elution buffer (2 × 5 mL), allowing incubations at 4 °C for 20 min per wash, and centrifuging at 2500× *g* and 4 °C between washes to pellet the resin. The eluates were dialyzed in Tris buffer (pH 7.0, 50 mM, 4 L) with magnesium chloride (20 mM) at 4 °C five times over 24 h, with a minimum of three hours per dialysis change. Protein expression and isolation were confirmed with SDS-PAGE Coomassie stained gel, which was examined for the band at the correct size, and a BCA assay to confirm purified protein concentration prior to use.

#### 3.2.2. CtHtrA Protease Inhibition Assay

CtHtrA was added to 25 wells in a black 96-well plate (40–80 µL, 10 µL increments), to which Tris-HCl buffer (50 mM, pH 7.0 with 20 mM MgCl_2_) was added to bring the total volume to 90 µL for 10 wells and 85 µL for the remaining 15. JO146 (10 µM, 5 µL) was added to 5 wells and JO146 (1 µM, 5 µL) was added to another 5 wells, with DMSO (5 µL) being added to the remaining 5 as a control. The plate was incubated at 37 °C for 10 min before substrate (MCA-ENLHLPLPIIFK-DNP, 10 µL, 1 mg/mL, 50% isopropanol in buffer) was added and analyzed in a TECAN Infinite Pro M200 plate reader with an excitation wavelength of 340 nm and fluorescence measured at 405 nm with four reads per well at 60 s intervals. A minimum volume of CtHtrA granting a fluorescence change of 300 min^−1^ was used for subsequent assay.

The determined appropriate volume of CtHtrA in buffer was added to a 96-well non-transparent black plate and diluted to 85 µL with buffer solution. Drug or control (5 µL) at a maximum of 20% DMSO/80% buffer was added to the appropriate well with DMSO controls, and the plate was incubated for 10 min to allow activation by the protein. MCA-ENLHLPLPIIFK-DNP (Mimotopes, Australia, 1 mg/mL, 10 µL) was added and immediately monitored on a TECAN M200 Infinite plate reader at 37 °C every 60 s for 30 min, using the same wavelengths as for the activity evaluation.

The rate of change of fluorescence was compared to controls to determine % inhibition of protein in Microsoft Excel^TM^, and these values were exported to GraphPad Prism for IC_50_ calculation. The positive control for **2a**–**g** was JO146, and (±)-**2g** was used as a positive control for **6a**–**h**.

#### 3.2.3. HLE Protease Inhibition Assay

Elastase was purchased from Athens Research and Technology (Cat No. 16-15-051200). Elastase (100 µg) was dissolved in Tris buffer (100 mM Tris, 20 mM CaCl_2_, pH 8.1, 3.14 mL), giving a concentration of 1.117 µM, and 25 µL was added to each well containing the respective inhibitor. The plate was incubated at 37 °C for 15 min.

*N*-Suc-AAA-pNA (Sigma Aldrich, Bayswater, Australia, S4760, 100 µL, 1 mg/mL, 45.1 µmol) was added to each well, and the absorbance was monitored every six minutes at 405 nm. The rate of change of absorbance was used as a comparison to controls to determine the % inhibition of protein in Microsoft Excel^TM^ (v2402), and these values were exported to GraphPad Prism (v8.0.1) for IC_50_ calculation.

#### 3.2.4. MTS Cell Viability Assay

HEp-2 (human epithelial type 2, CCL-23, ATCC^®^_,_ Virginia, USA) or McCoy B (mouse fibroblast, CRL-1696, ATCC^®^) cells were grown to 80–100% confluence, and cell density was determined with a haemocytometer. Cells were then diluted to 1 × 10^5^ cells/mL, and 100 µL was added to each well in a 96-well plate, with 3 wells left as controls. Cells were grown for 24 h, and the media was removed and then replaced with fresh media containing 0.1% DMSO and the drug for testing in triplicate. Drugs and cells were incubated for 24 h, and then 20 µL of MTS and PMS solution was added and then incubated for a further four hours. Absorbance was measured at 490 nm on a TECAN M200 Infinite plate reader and data were processed using Microsoft Excel^TM^ with the following equation to determine cell viability relative to DMSO controls.

$$Cell\;viability\;\left(\%\right)=\frac{{Absorbance}_{Drug}-{Absorbance}_{Dye}}{{Absorbance}_{DMSO}-{Absorbance}_{Dye}}$$

#### 3.2.5. *Chlamydia trachomatis* Growth Inhibition Assay

HEp-2 cells were grown until confluent in a T-75 flask with DMEM (containing 10% FCS, gentamycin (0.05 mg/mL), streptomycin (0.1 mg/mL) and glutamine (Sigma-Aldrich Cat No. G7513, 4 mM); they were then harvested with trypsin, and the cell density was determined with a haemocytometer. Cells were diluted to 1 × 10^5^ cells/mL, and 200 µL was dispensed to each well in a 96-well plate and then incubated for 24 h at 5% CO_2_ at 37 °C.

The supernatant was removed, and 100 µL of the respective chlamydia species in media (100,000 IFU/mL) was added, giving an MOI of 0.5. The plate was sealed and centrifuged at 500× *g*/28 °C for 30 min and then incubated for a further 3.5 h at 37 °C with 5% CO_2_. The supernatant was removed, and 100 µL of cycloheximide in DMEM (1 µg/mL) was added in addition to 100 µL of drug stock solution to each respective well with DMSO and media negative controls and also azithromycin positive control. The plate was incubated for a further 26 h.

The supernatant was removed, and wells were washed with PBS (2 × 150 µL) and then fixed in methanol for eight minutes. The methanol was removed and washed with additional PBS (2 × 150 µL) and stored at 4 °C with 100 µL PBS per well and then stained and analyzed using fluorescence microscopy, which is outlined below.

Cells in fixed plates were permeabilized with 0.5% Triton X-100 in PBS for 15 min and then blocked with 1% BSA in PBS for 45 min. Anti-HtrA polyclonal antibody (sera) (1:600) (in-house [8]), anti-mouse tubulin (Sigma-Aldrich Cat no. T-5168, 1:3000) and DAPI (125 ng/mL) in PBS were added to each well (70 µL) and then gently shaken at room temperature while shielding it from light for 45 min. The primary antibodies were removed, and the wells were washed with PBS (4 × 150 µL). Secondary antibodies (goat anti-rabbit Alexafluor 488, Thermo Fisher Cat No. A-11008 and goat anti-mouse Alexafluor 568 (Thermo Fisher Cat No. A-11004)) in PBS (70 µL, 1:600 respectively) were added and the wells were gently shaken at room temperature while shielding them from light for 45 min. The antibodies were removed, and the wells were washed with PBS (5 × 150 µL); cells were then stored with PBS (100 µL per well) at 4 °C pending analysis.

Plates were analyzed on an INcell Analyzer 2200 (Cytiva Life Sciences, Marlborough, MA, USA) using both laser autofocus (10%) and software autofocus modes, with ten random images per well covering a minimum of 80% of the well at 10x magnification. Non-morphologically affected inclusions were counted using a macro in ImageJ (v1.54f), and MIC was defined as being one two-fold dilution more concentrated than MIC_TP_ (where MIC_TP_ is defined as the lowest concentration to produce ≥90% morphologically abnormal inclusions) relative to solvent controls [27].

## 4. Conclusions

In this work, we explored the structure–activity relationships of 3-alkoxy-4-chloroisocoumarins as serine protease inhibitors against CtHtrA and HLE. The compounds were further evaluated for their cytotoxic properties against HEp-2 and McCoy B cells and for their anti-chlamydial properties against *C. trachomatis*. Steric bulk on the C-3 alkoxy chain was identified as the driving factor for CtHtrA inhibitory potency and selectivity between the two proteases, while C-7 substitution was found to reduce the inhibitory potency against CtHtrA. Compounds with C-7 substituents were slightly more cytotoxic than their unsubstituted analogue against HEp-2 and McCoy B cells. It was found that 7-amino and 7-dimethylamino derivatives afford 4-chloroisocoumarin compounds with anti-chlamydial properties. However, these anti-chlamydial properties were not found to correlate with their CtHtrA inhibitory activity.

## Figures and Tables

**Table 1:** Inhibitory activity of 4-chloroisocoumarins against CtHtrA and HLE in protease inhibition assays relative to JO146 (**2a–2g**) or **2g** (**6a–6h**).

Compound	Relative Potency against CtHtrA ^a^	Relative Potency against HLE ^b^	Relative Selectivity ^c^
JO146	1.00	1.00	1.00
**2a**	2.33	0.0207	113.0
**2b**	3.73	0.0664	56.4
**2c**	2.23	0.323	6.92
**2d**	1.57	0.310	5.08
**2e**	5.32	0.419	12.7
**2f**	1.50	0.368	4.08
**2g**	6.58	0.451	14.6
**6a**	0.326	n.d.	n.d.
**6b**	0.113	n.d.	n.d.
**6c**	0.158	n.d.	n.d.
**6d**	<0.001	n.d.	n.d.
**6e**	<0.001	n.d.	n.d.
**6f**	0.057	n.d.	n.d.
**6g**	0.390	n.d.	n.d.
**6h**	0.184	n.d.	n.d.

^a^ Relative potency is given as [IC_50_ of control*]/[IC_50_ test compound]; ^b^ relative potency is given as [IC_50_ of JO146]/[IC_50_ test compound]; ^c^ relative selectivity is given as (relative CtHtrA potency)/(relative HLE potency)* ; n.d. = not determined.

## Data Availability

All data are either presented in the body of this article or in the Supplementary Materials.

## Supplementary Materials

The following supporting information can be downloaded at: https://www.mdpi.com/article/10.3390/molecules29071519/s1, Optical absorbance of **2a–2g** and **6a–h** (Figure S1); IC_50_ data and dose–response curves used in protease inhibition assays (Figures S2–S4 and Tables S1 and S2); substituent hydrophobicity values (Table S3); cytotoxicity of **2a–g** and **6a–h** against McCoy B cells (Figure S5); workflow description for anti-chlamydial assays (Figure S6); representative qNMR spectra for compounds **2g** and **6a** (Figures S7 and S8); quantitative NMR data for compounds tested in biological assays (Table S3); ^1^H and ^13^C NMR spectra for newly synthesized compounds and compounds whose NMRs are not previously reported (Figures S9–S46).
