# Supplementary material for: 4-Chloroisocoumarins as Chlamydial Protease Inhibitors and Anti-Chlamydial Agents
Source: Molecules. 2024 Mar 28;29(7):1519. doi: 10.3390/molecules29071519 (PMC11013143; doi:10.3390/molecules29071519)
Supplement: Supplementary file 1 [file molecules-29-01519-s001.zip › molecules-2917384-supplementary.pdf]

Supplementary Information

4-Chloroisocoumarins as Chlamydial Protease Inhibitors and Anti-Chlamydial Agents

Matthew J. A. Phillips [1], Wilhelmina M. Huston [2], Andrew M. McDonagh [1,*] and Tristan Rawling [1,*]

[1] School of Mathematical and Physical Sciences, Faculty of Science, University of Technology Sydney, Sydney, NSW 2007, Australia; matthew.phillips-1@uts.edu.au
[2] School of Life Sciences, Faculty of Science, University of Technology Sydney, Sydney, NSW 2007, Australia; wilhelmina.huston@uts.edu.au
* Correspondence: andrew.mcdonagh@uts.edu.au (A.M.M.); tristan.rawling@uts.edu.au (T.R.)

Table of contents

1.1 Optical absorbance of **2a-g** and **6a-h**.. 2

1.2 Representative dose-response curves of HNE protease inhibition assays 3

1.3 Representative dose-response curves of CtHtrA protease inhibition assays........................ 4

1.4 Half-maximal inhibitory concentration values (IC_{50}) of inhibitors against HLE 6

1.5 Half-maximal inhibitory concentration values (IC_{50}) of inhibitors against CtHtrA 6

1.6 Substituent hydrophibicity values 7

1.7 Cytotoxic effects of **2a-2g** and **6a-6h** against McCoy B cells..7

1.8 Workflow for the evaluation of anti-chlamydial properties of **2a-g** and **6a-h**..............................8

1.9 Quantification of compound purity by ^{1}H NMR of **2a-g** and **6a-h** 9

1.10 ^{1}H NMR and ^{13}C NMR Spectra..11

References.. 30

1.1. Optical Absorbance of ***2a-g*** *and* ***6a-h***

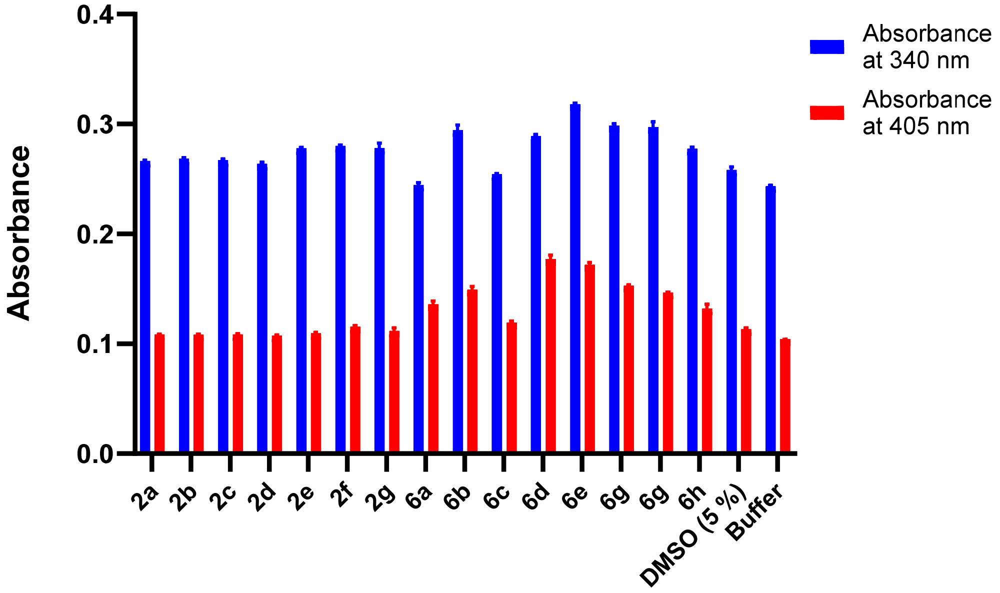

Figure S1. Absorbance of **2a-g** and **6a-h** at 50 μM in 50 mM Tris, 20 mM $MgCl_2$, pH 7.0 at 340 nm and 405 nm. Error bars show the standard deviation from n = 3 analyses.

1.2. Representative Dose-Response Curves of HNE Protease Inhibition Assays

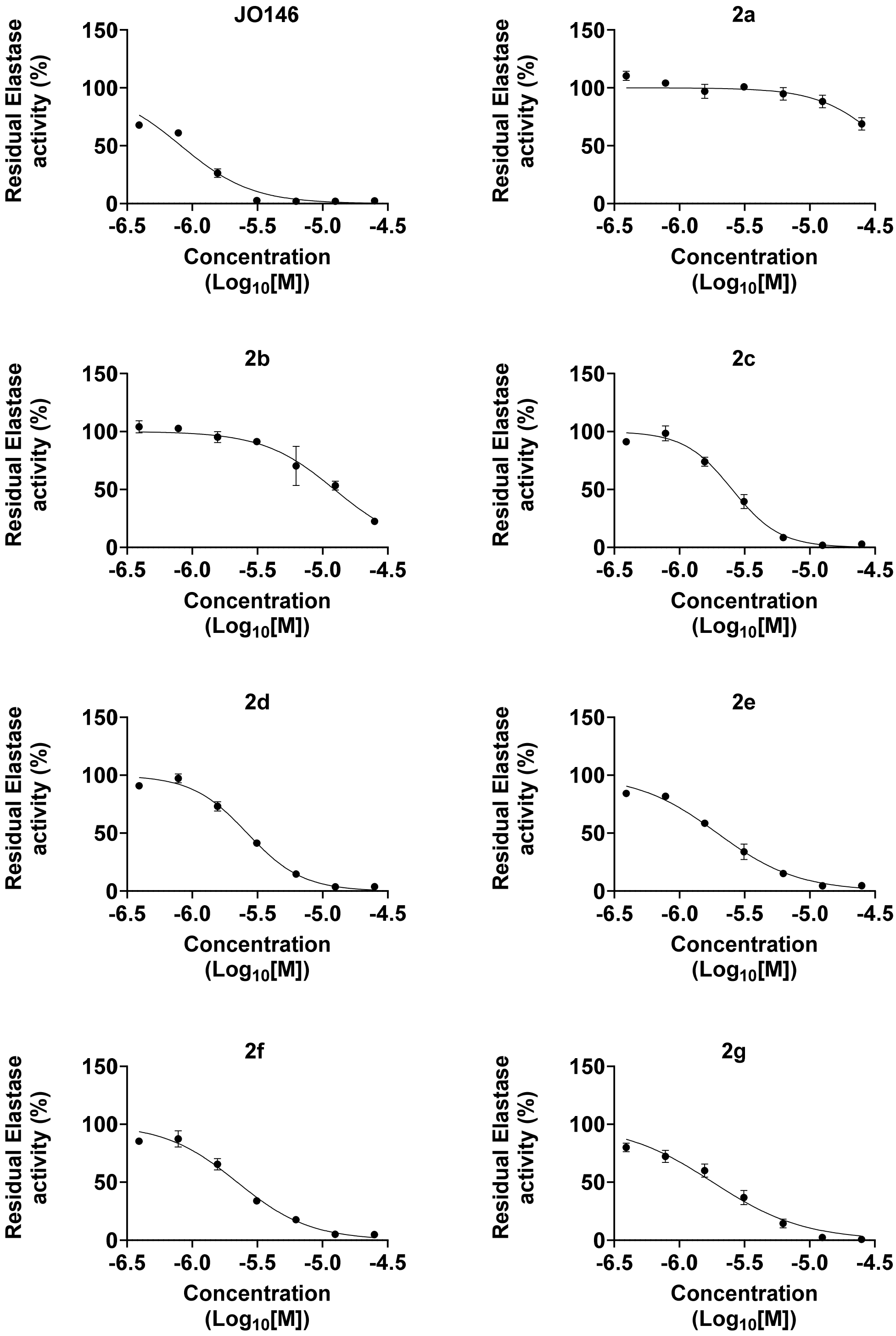

Figure S2. Dose-response curves of residual HLE activity against compounds **2a**-**g** and JO146.

1.3. Representative Dose-Response Curves of CtHtrA Protease Inhibition Assays

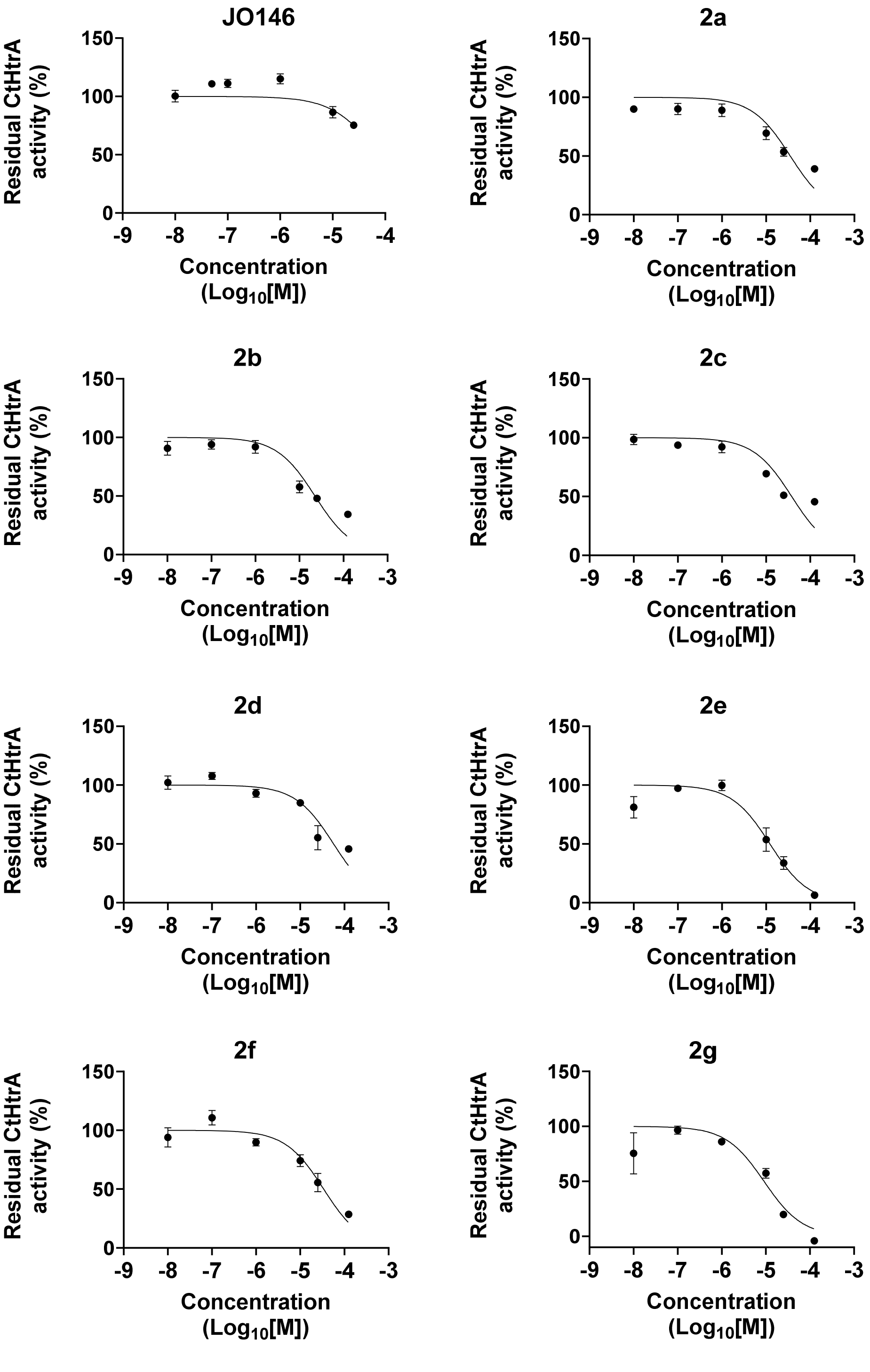

Figure S3. Dose-response curves of residual CtHtrA activity against compounds **2a**-**g** and JO146. Error bars represent the geometric mean from three replicates.

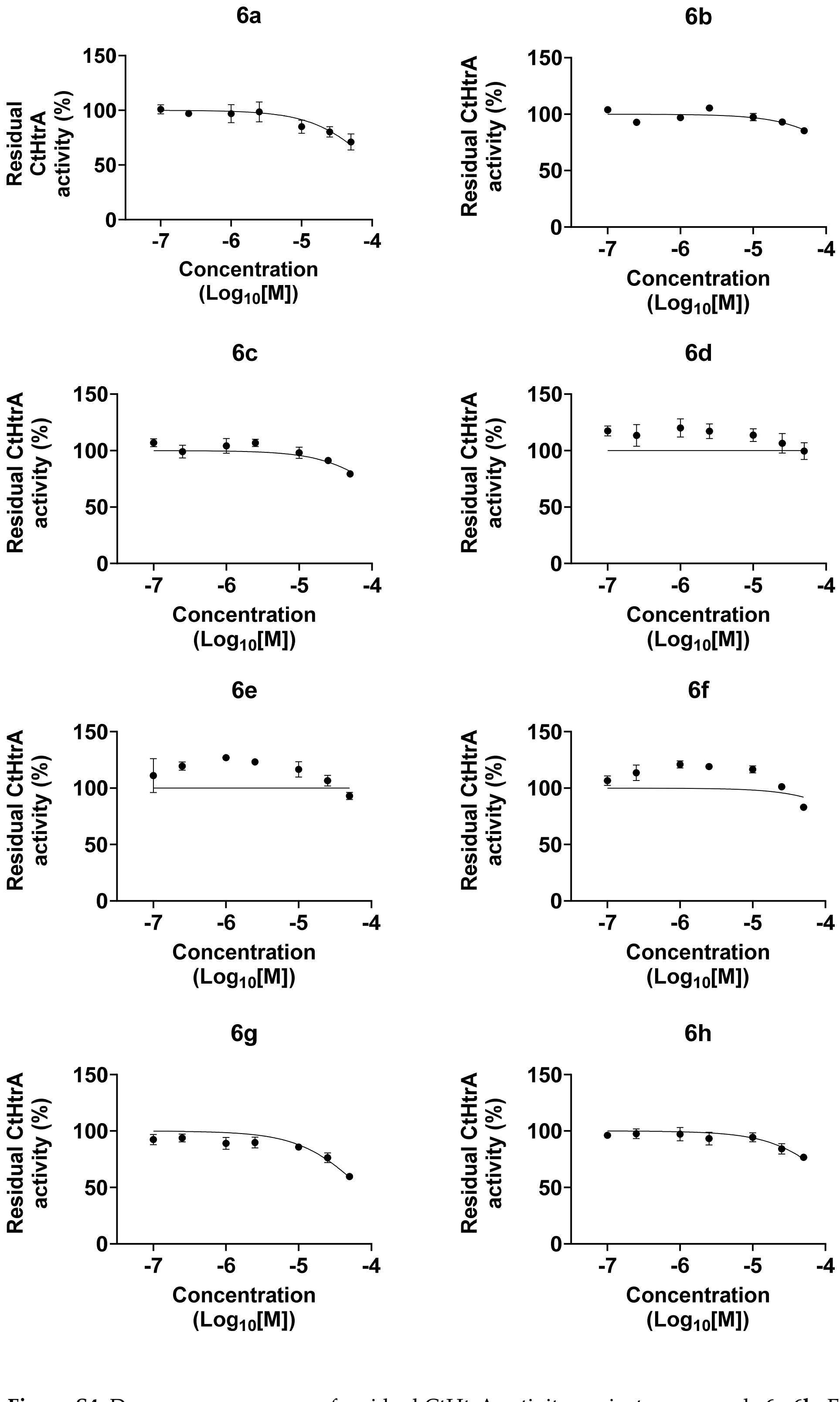

Figure S4. Dose-response curves of residual CtHtrA activity against compounds **6a**-**6h**. Error bars represent the geometric mean from three replicates.

1.4. Half-Maximal Inhibitory Concentration Values (IC_{50}) of Inhibitors against HLE

Table S1. IC_{50} data, 95 % confidence intervals and correlation values for protease inhibition assays against HLE.

Compound	IC_{50} (μM)	95 % CI	R^2 value
JO146	0.817	0.713-0.929	0.9527
**2a**	39.5	30.6-65.2	0.8005
**2b**	12.3	10.7-14.3	0.9446
**2c**	2.53	2.34-2.75	0.9852
**2d**	2.64	2.46-2.85	0.9896
**2e**	1.95	1.76-2.16	0.9873
**2f**	2.22	2.00-2.45	0.9792
**2g**	1.81	1.57-2.07	0.9659

IC_{50} values calculated based on log(inhibitor) vs. normalized response-Variable slope (four parameter) using Graphpad Prism software (version 8.0.1). Model fitness is represented in R^2 values.

1.5. Half-Maximal Inhibitory Concentration Values (IC_{50}) of Inhibitors against CtHtrA

Table S2. IC_{50} data, 95 % confidence intervals and correlation values for protease inhibition assays against CtHtrA.

	Compound	IC_{50} (μM)	95 % CI	R^2 value
**Batch 1**	**JO146 (control)**	71.5	38.7-184	0.3991
	**2a**	30.6	17.4-56.1	0.4945
	**2b**	19.1	11.6-31.8	0.7345
	**2c**	32.0	17.0-64.1	0.4022
	**2d**	45.5	26.7-80.6	0.7323
**Batch 2**	**JO146 (control)**	60.0	32.6-148	0.4990
	**2e**	11.3	6.15-20.0	0.8180
	**2f**	40.0	26.3-61.5	0.8713
	**2g**	9.11	4.77-16.2	0.8081
**Batch 3**	**2g (control)**	34.9	25.5-48.5	0.8387
	**6a**	107	82.7-144	0.7331
	**6b**	309	222-482	0.5674
	**6c**	221	157-347	0.6588
**Batch 4**	**2g (control)**	32.3	19.9-54.7	0.7469
	**6d**	Not active	-	-1.952
	**6e**	Not active	-	-1.321
	**6f**	568	-	-0.3867
**Batch 5**	**2g (control)**	28.0	20.2-39.3	0.8632
	**6g**	71.8	56.2-93.3	0.6756
	**6h**	152		0.6884

IC_{50} values calculated based on log(inhibitor) vs. normalized response (three parameter) using Graphpad Prism software (version 8.0.1).

1.6. Substituent Hydrophibicity Values

Table S3. Substituent hydrophobicity parameters (π) for C-7 isocoumarins as reported by Hansch *et al.* [26].

Functional group	π	σ_m	σ_p	Molar refractivity
H	0.00	0.00	0.00	1.03
NH_2	-1.23	-0.16	-0.66	5.42
$N(CH_3)_2$	0.18	-0.15	-0.83	15.55
$NHCOCH_3$	-0.97	0.21	0.00	14.93
$NHCOCF_3$	0.08	0.20	0.03	18.17
I	1.12	0.35	0.18	13.94
Br	0.86	0.39	0.23	8.88
Cl	0.71	0.37	0.23	6.03
OCH_3	-0.02	0.12	-0.27	7.87

*1.7. Cytotoxic effects of **2a-2g** and **6a-6h** against McCoy B cells*

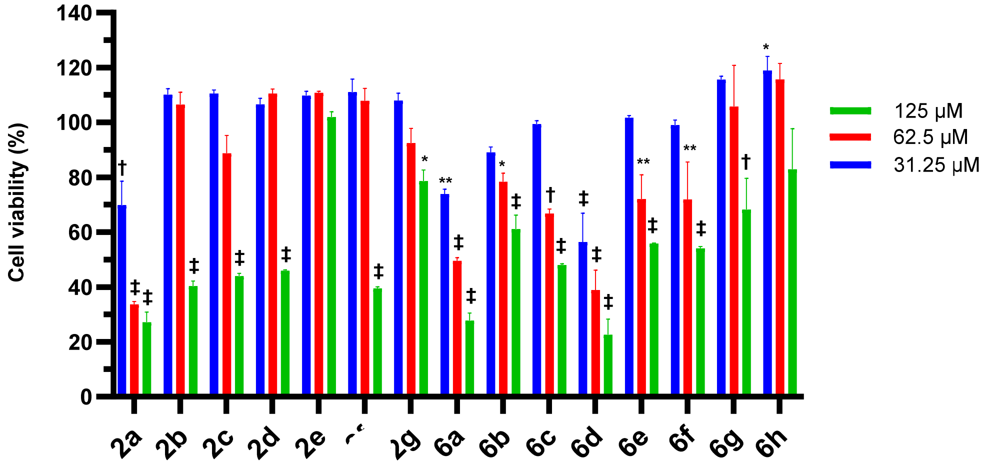

Figure S5. Cytotoxicity of **2a**-**g** and **6a**-**h** against McCoy B cells (24 h, 31.25 μM, 62.5 μM and 125 μM). Values represent the average ± SEM from three independent experiments. Significance shown is relative to solvent (0.1 % DMSO) controls: (*) $p \leq 0.05$, (**) $p \leq 0.01$, (†) $p \leq 0.005$, (‡) $p \leq 0.001$.

1.8. Workflow for the evaluation of anti-chlamydial properties of ***2a-g*** *and* ***6a-h***

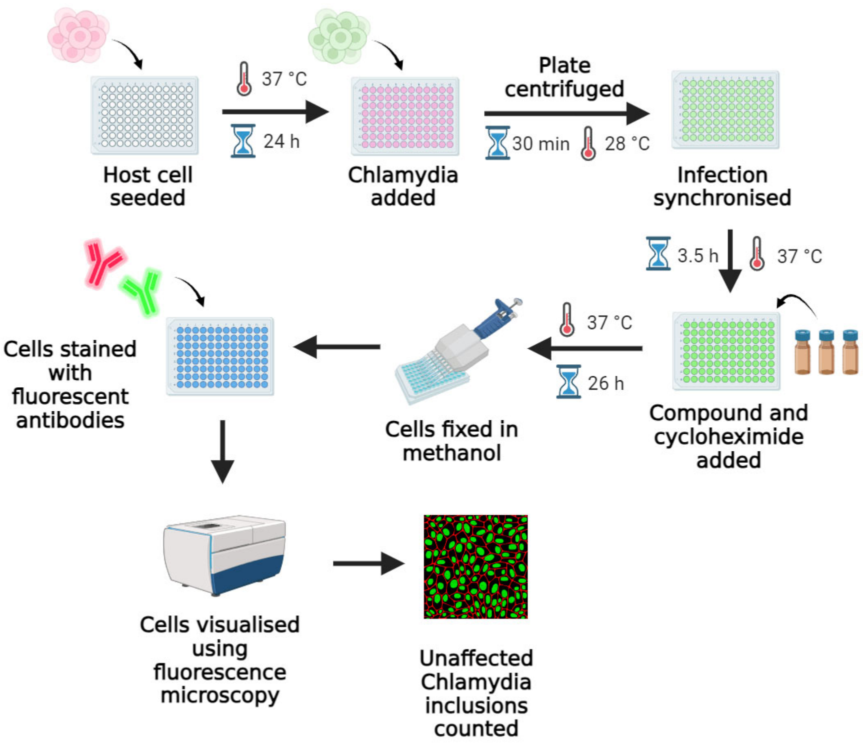

Figure S6. Workflow for the evaluation of anti-chlamydial properties of **2a**-**g** and **6a**-**h**

1.9. *Quantification of compound purity by ^{1}H NMR of* ***2a-g*** *and* ***6a-h***

The purity of compounds tested in biological assays were confirmed to be ≥ 95 % by quantitative ^{1}H NMR spectroscopy (qNMR) using the procedure detailed in the Journal of Medicinal Chemistry [28,29]. This technique uses a spiked NMR sample of known quantity with an internal standard of a known purity, and the integrals of these signals are compared. For all samples, d_6-DMSO was spiked with 1,3,5-trioxane (99.5 % purity) to give a known concentration between 3-5 mg/mL. Between 5-10 mg (mass weighed to 5 decimal places on an analytical scale) was dissolved in 600 µL of the spiked d_6-DMSO solution, then transferred to a 5 mm NMR tube. Parameters used for ^{1}H NMR collection, processing and the calculation of purity followed those reported in the Journal of Medicinal Chemistry[29]. The calculated purity of each tested compound is shown in Table S1. Exemplar qNMR spectra for compound **2g** (Figure S7) and **6a** (Figure S8) are provided below.

Table S3. Purity of 4-chloroisocoumarin compounds tested in biological assays as determined by qNMR.

Compound	Purity (%)
**2a**	98.9
**2b**	96.2
**2c**	101.8
**2d**	99.8
**2e**	102.4
**2f**	99.2
**2g**	97.5
**6a**	97.2
**6b**	97.0
**6c**	97.4
**6d**	97.9
**6e**	100.3
**6f**	97.2
**6g**	97.4
**6h**	95.3

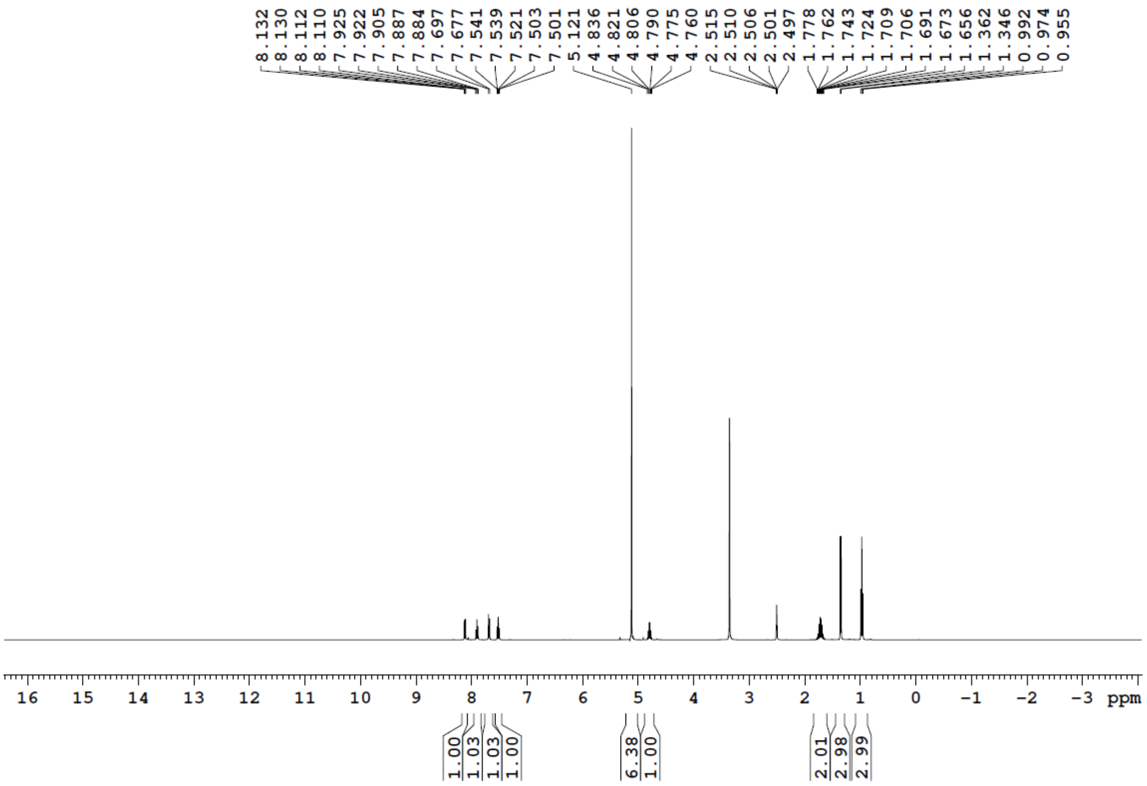

Figure S7. Representative quantitative ^{1}H NMR spectrum of **2g** in *d*$_6$-DMSO spiked with 1,3,5-trioxane.

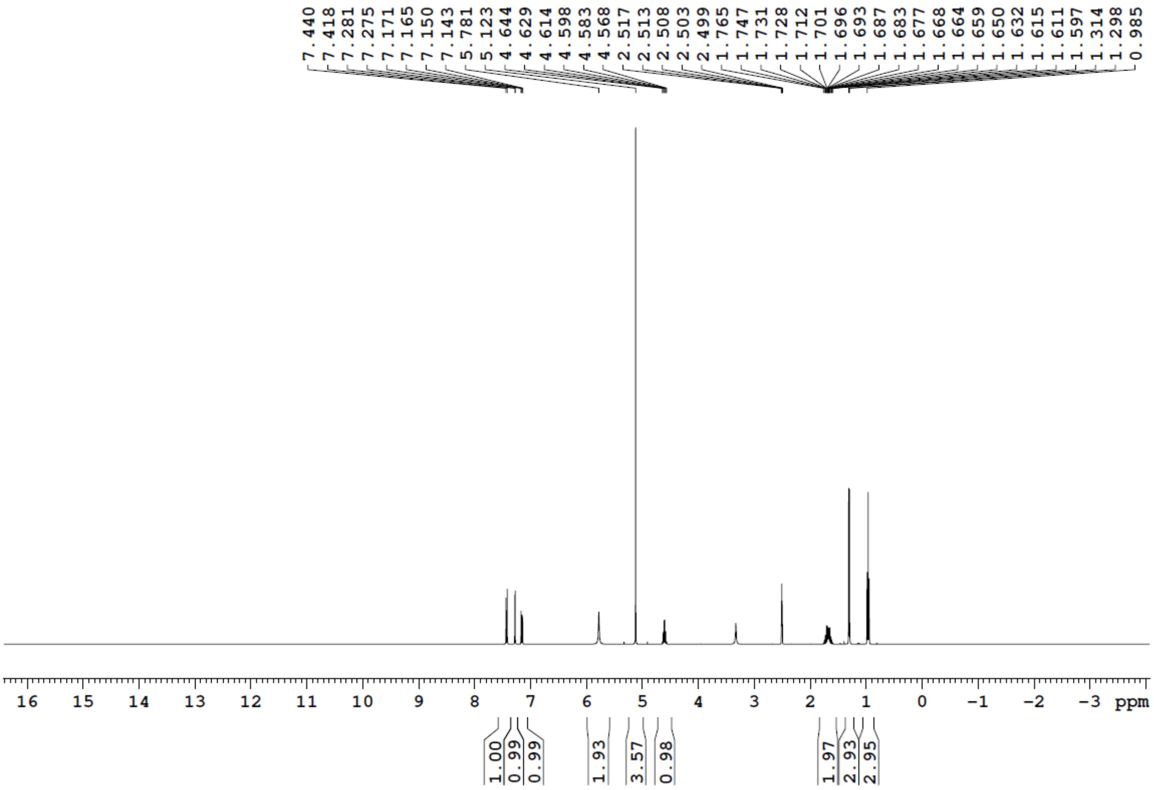

Figure S8. Representative quantitative ^{1}H NMR spectrum of **6a** in *d*$_6$-DMSO spiked with 1,3,5-trioxane.

1.10. ¹H NMR and ¹³C NMR Spectra

2-(2-*iso*-Butoxy)-2-oxo-ethyl)benzoic acid (**1f**)

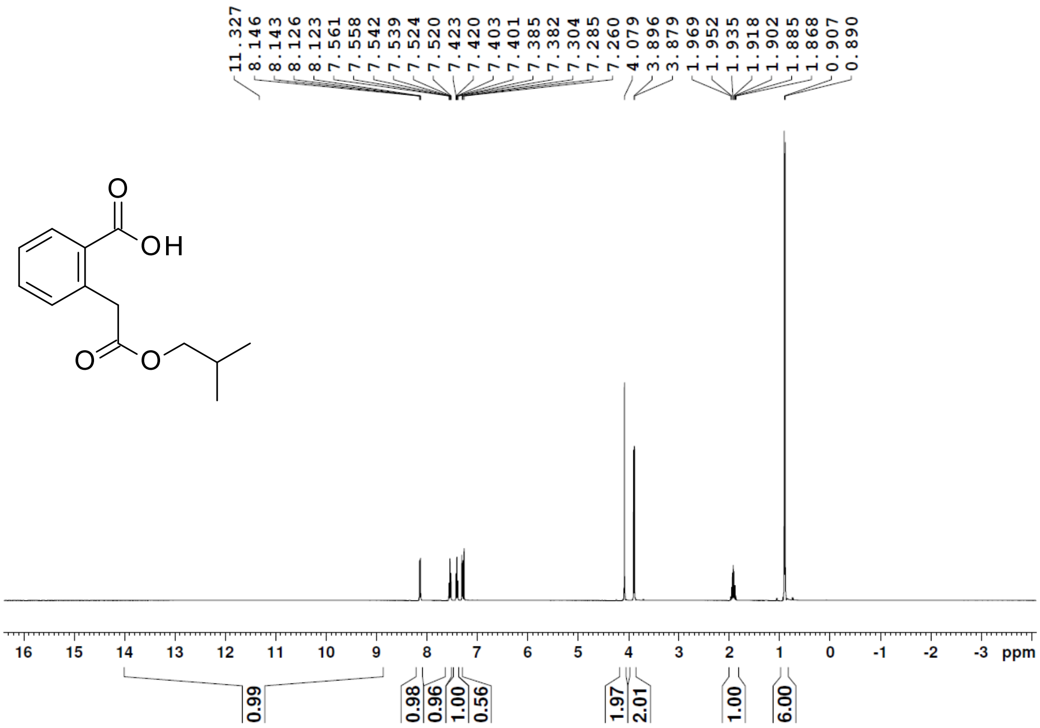

Figure S9. The 400 MHz ^{1}H NMR spectrum of **1f** in $CDCl_3$.

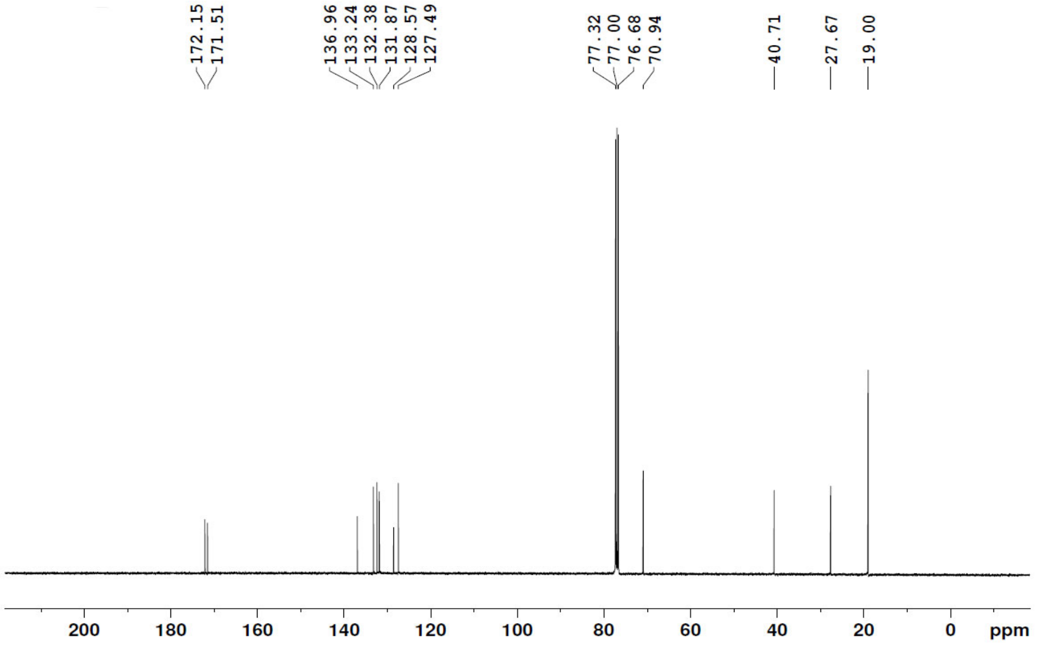

Figure S10. The 100 MHz ^{13}C NMR spectrum of **1f** in $CDCl_3$.

(±)-2-(2-*sec*-Butoxy)-2-oxo-ethyl)benzoic acid (**1g**)

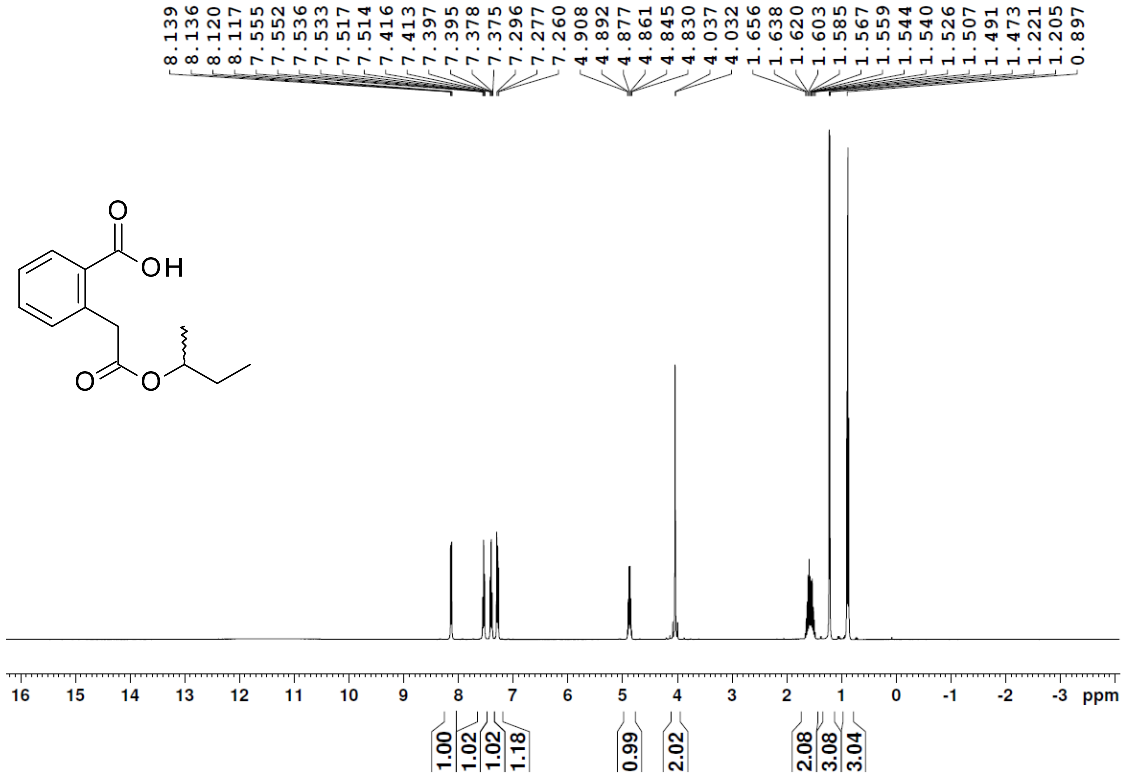

Figure S11. The 400 MHz ^1H NMR spectrum of **1g** in $CDCl_3$.

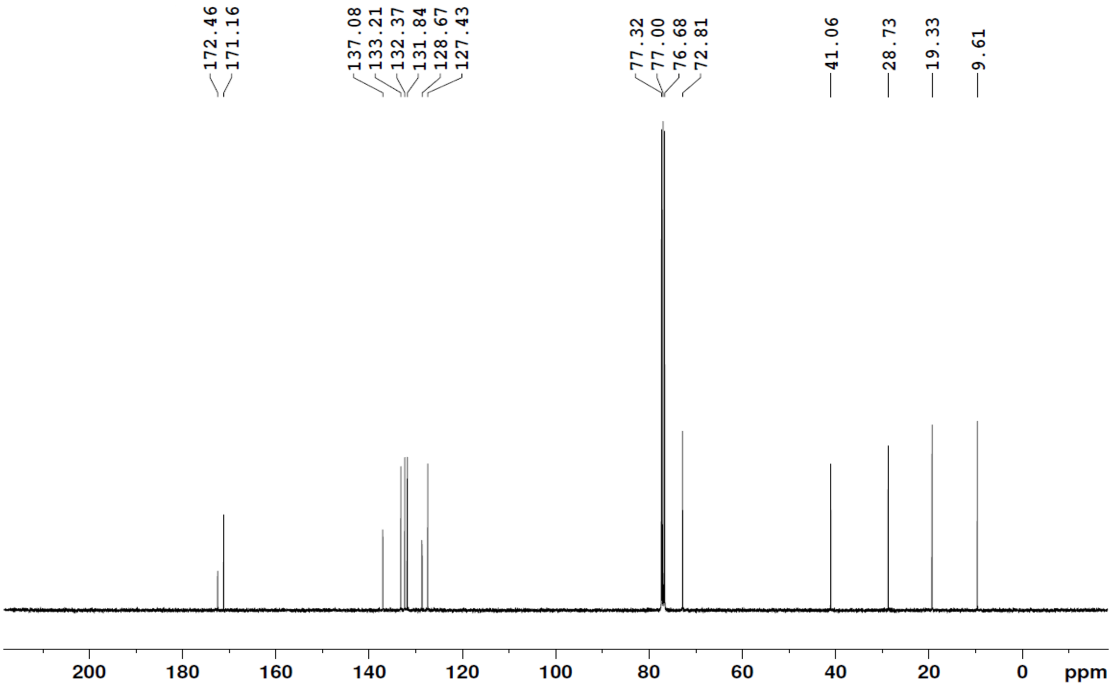

Figure S12. The 100 MHz ^{13}C NMR spectrum of **1g** in $CDCl_3$.

(±)-5-Chloro-2-(2-*sec*-butoxy-2-oxo-ethyl)benzoic acid (**9**)

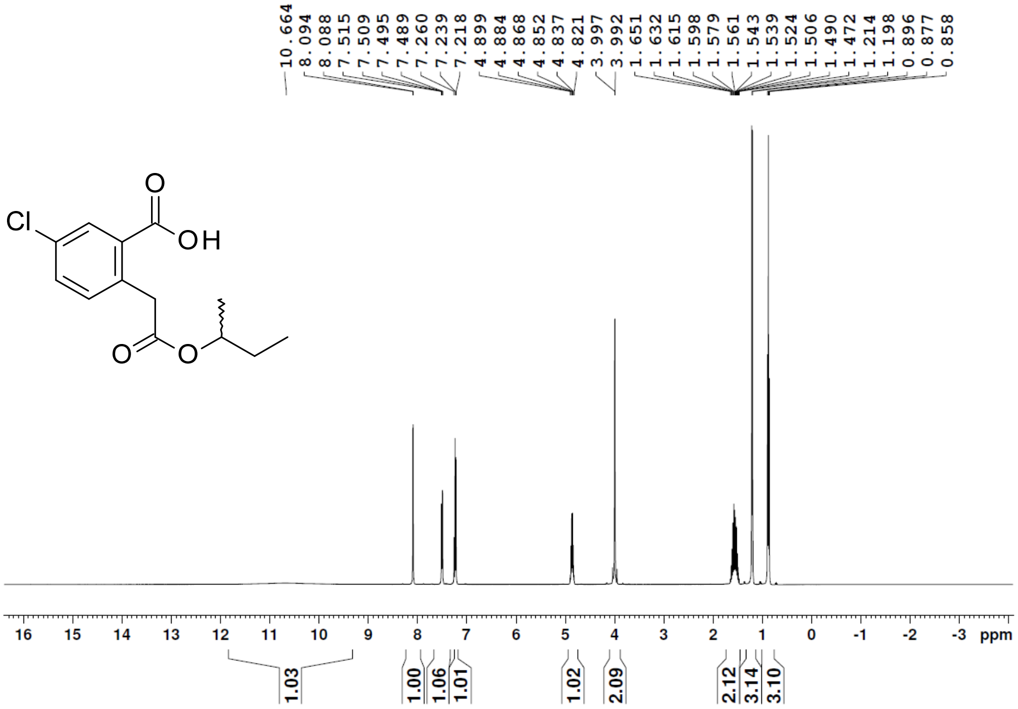

Figure S13. The 400 MHz ^{1}H NMR spectrum of **9** in $CDCl_3$.

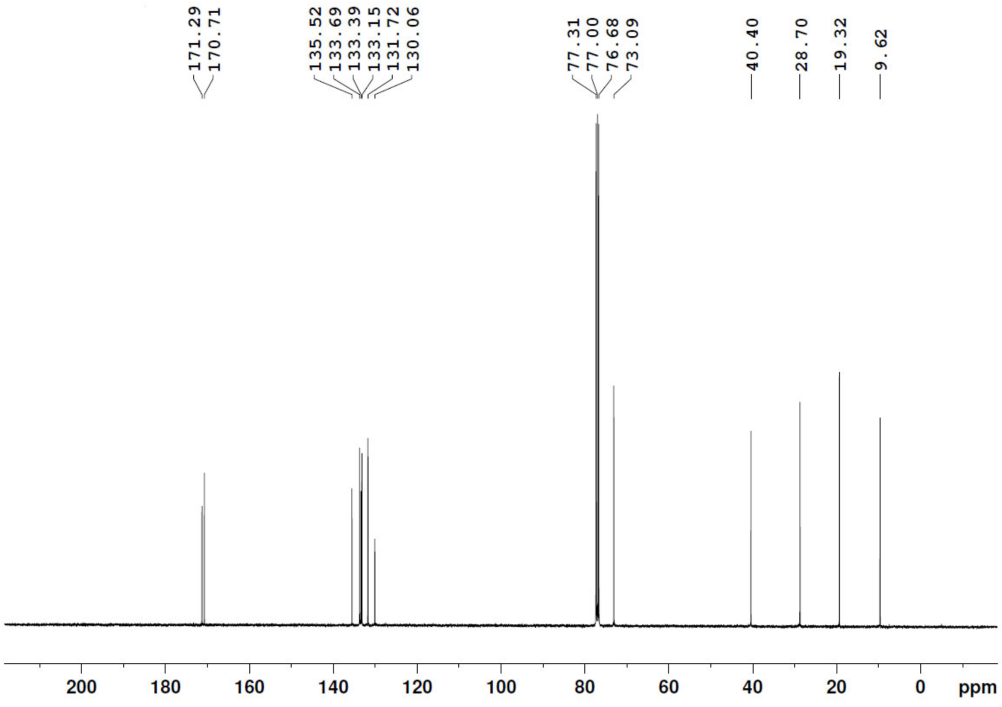

Figure S14. The 100 MHz ^{13}C NMR spectrum of **9** in $CDCl_3$.

(±)-5-Methoxy-2-(2-*sec*-butoxy-2-oxo-ethyl)benzoic acid (**14**)

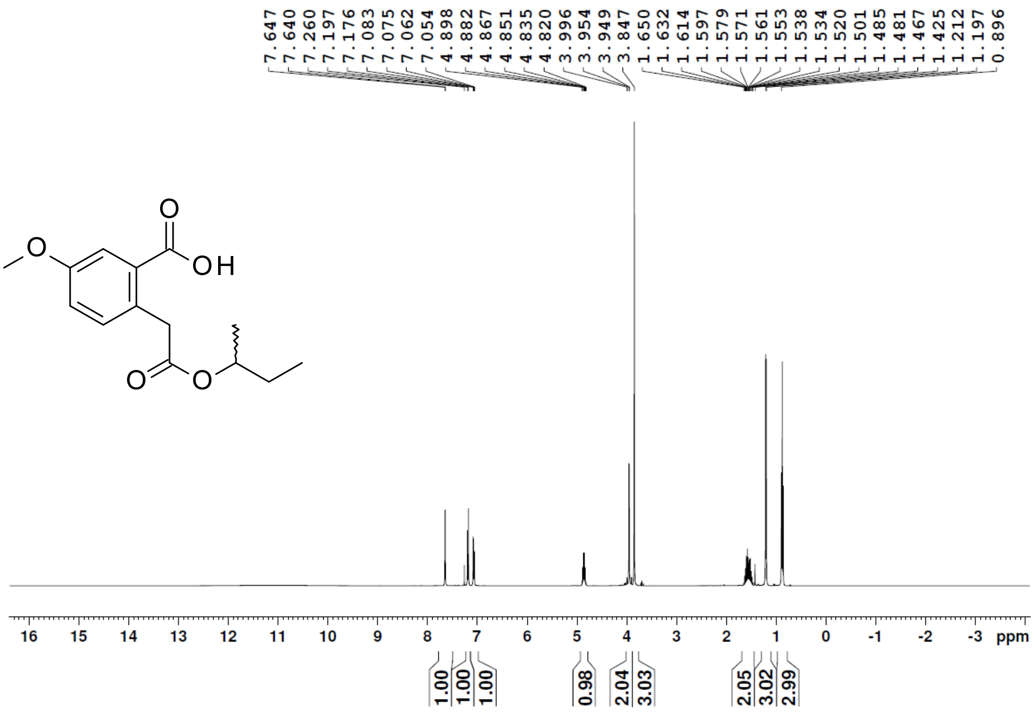

Figure S15. The 400 MHz ^{1}H NMR spectrum of **14** in $CDCl_3$.

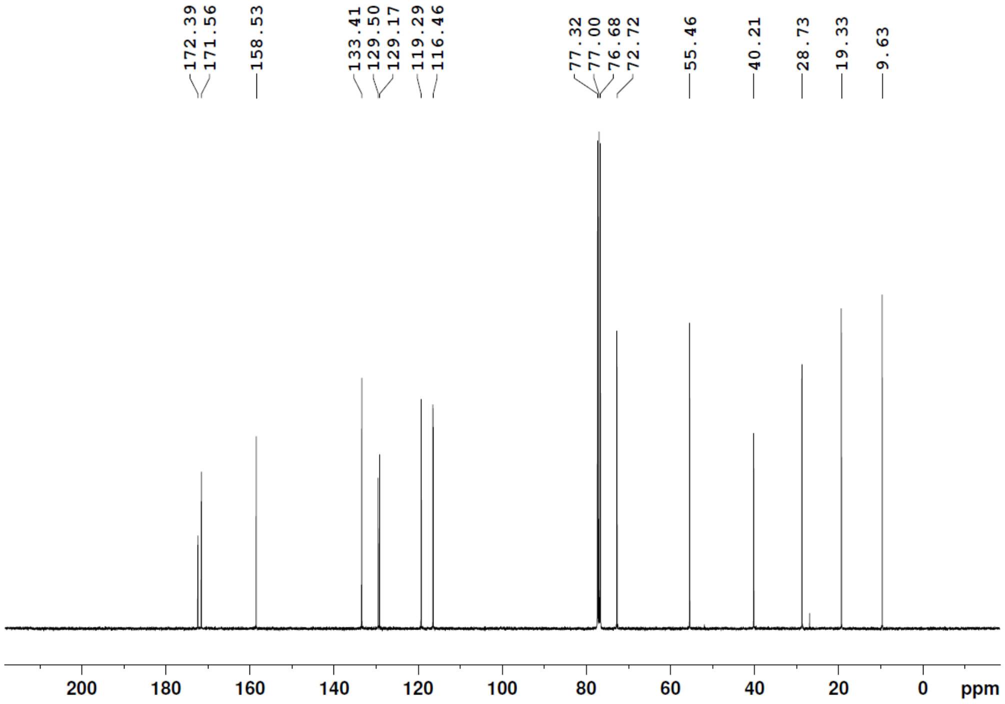

Figure S16. The 100 MHz ^{13}C NMR spectrum of **14** in $CDCl_3$.

3-Methoxy-4-chloroisocoumarin (**2a**)

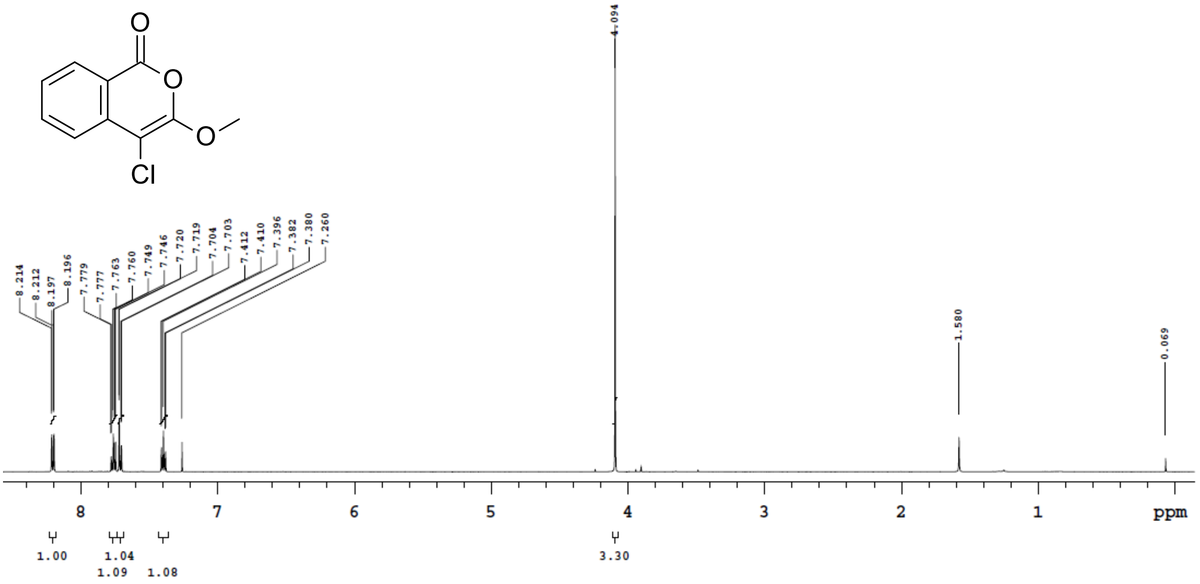

Figure S17. The 500 MHz ^{1}H NMR spectrum of **2a** in $CDCl_3$.

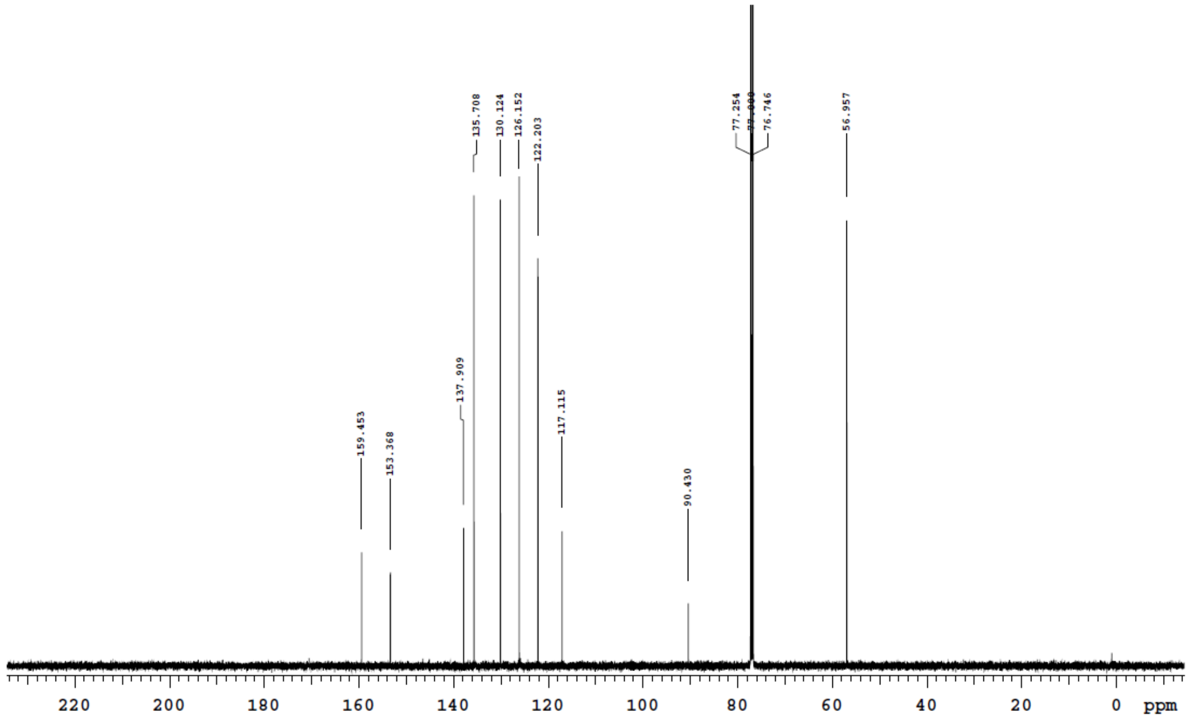

Figure S18. The 125 MHz ^{13}C NMR spectrum of **2a** in $CDCl_3$.

3-Ethoxy-4-chloroisocoumarin (**2b**)

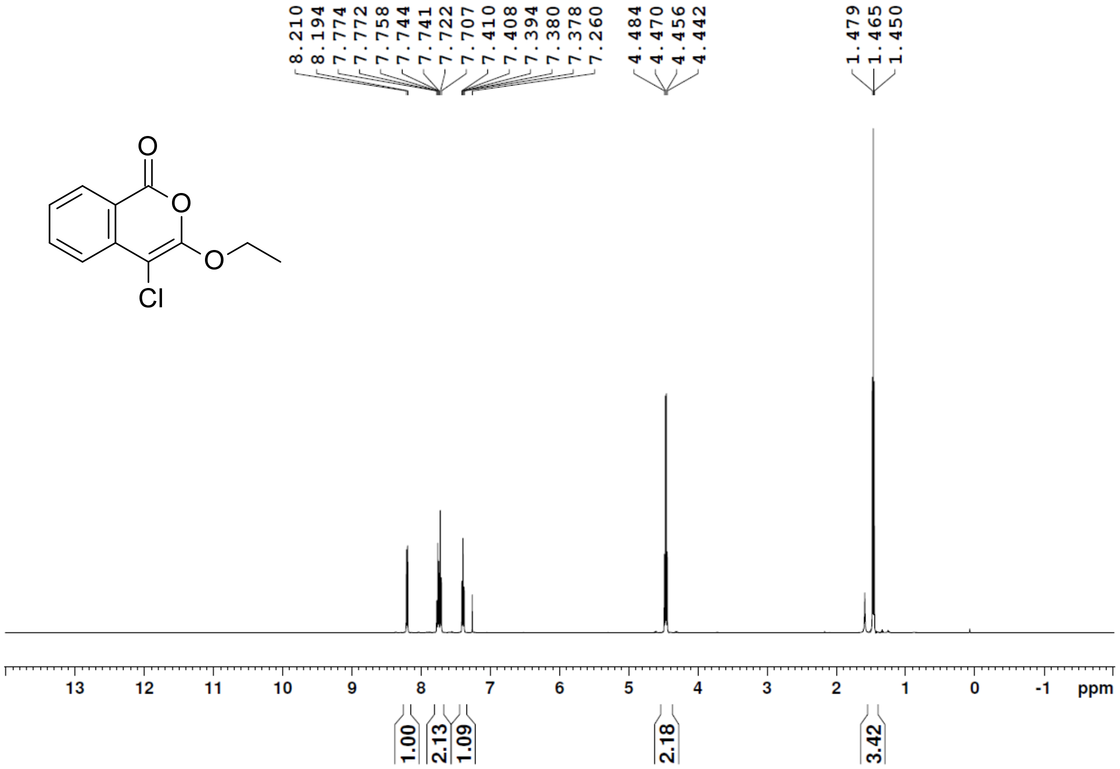

Figure S19. The 400 MHz ^{1}H NMR spectrum of **2b** in $CDCl_3$.

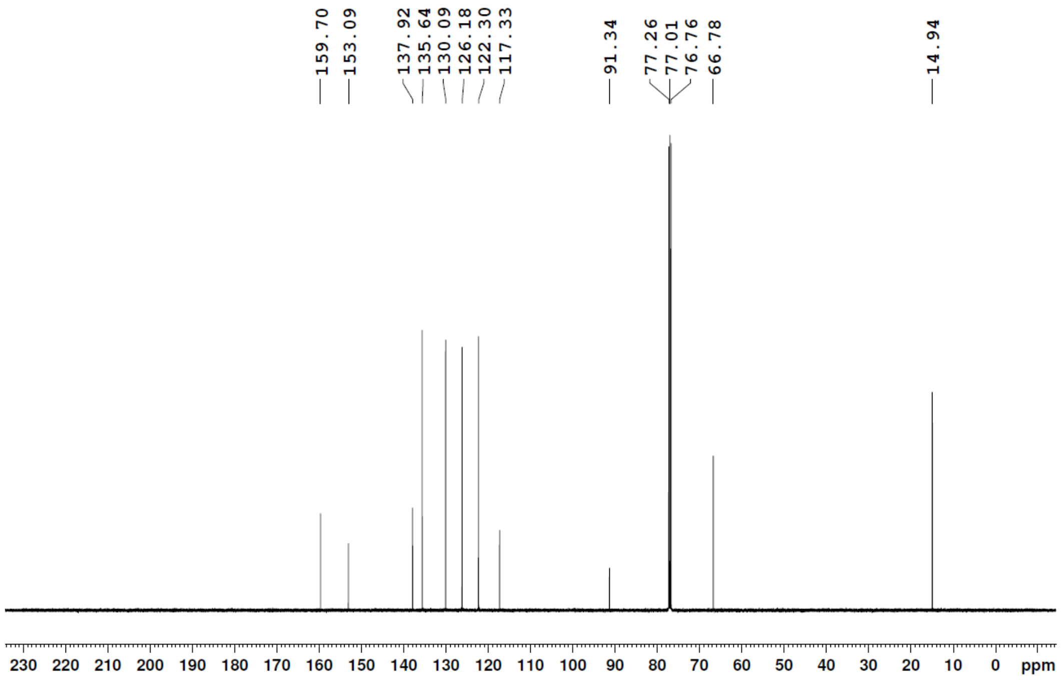

Figure S20. The 100 MHz ^{13}C NMR spectrum of **2b** in $CDCl_3$.

3-*iso*-Propoxy-4-chloroisocoumarin (**2e**)

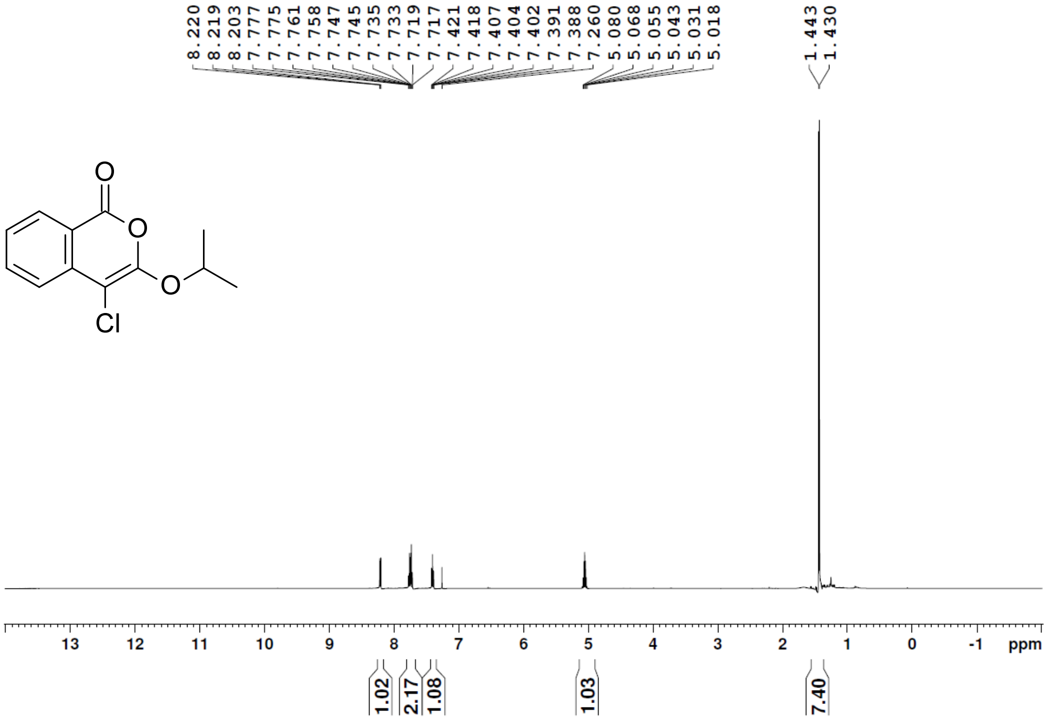

Figure S21. The 400 MHz ^{1}H NMR spectrum of **2e** in $CDCl_3$.

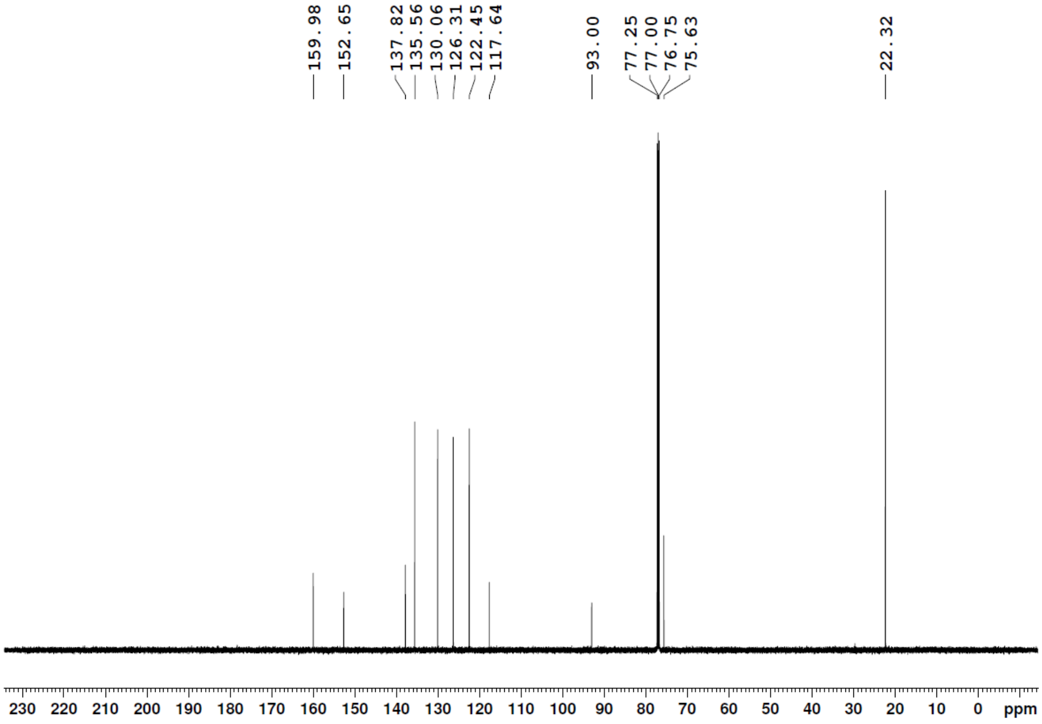

Figure S22. The 100 MHz ^{13}C NMR spectrum of **2e** in $CDCl_3$.

3-*iso*-Butoxy-4-chloroisocoumarin (**2f**)

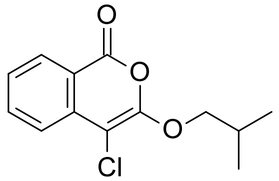

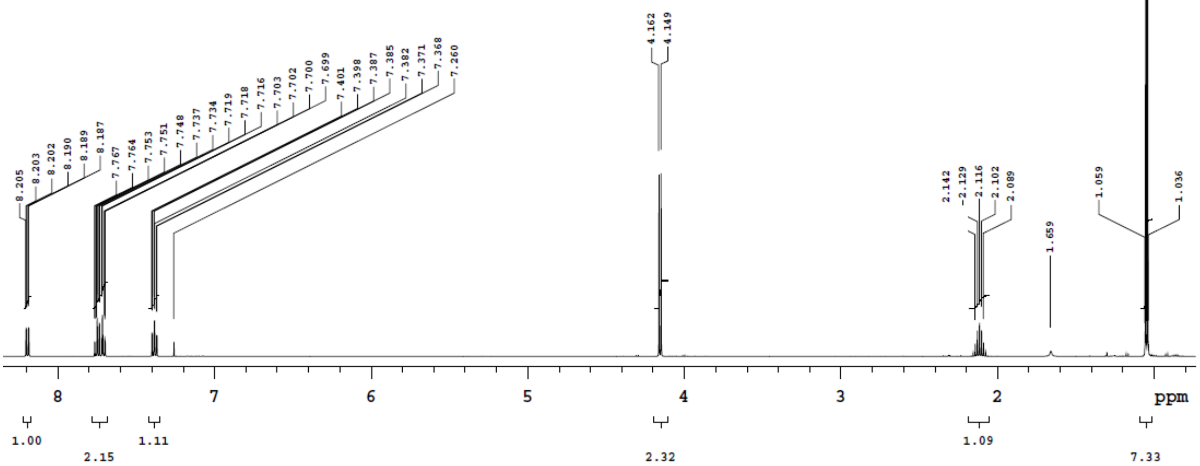

Figure S23. The 500 MHz ^{1}H NMR spectrum of **2f** in $CDCl_3$.

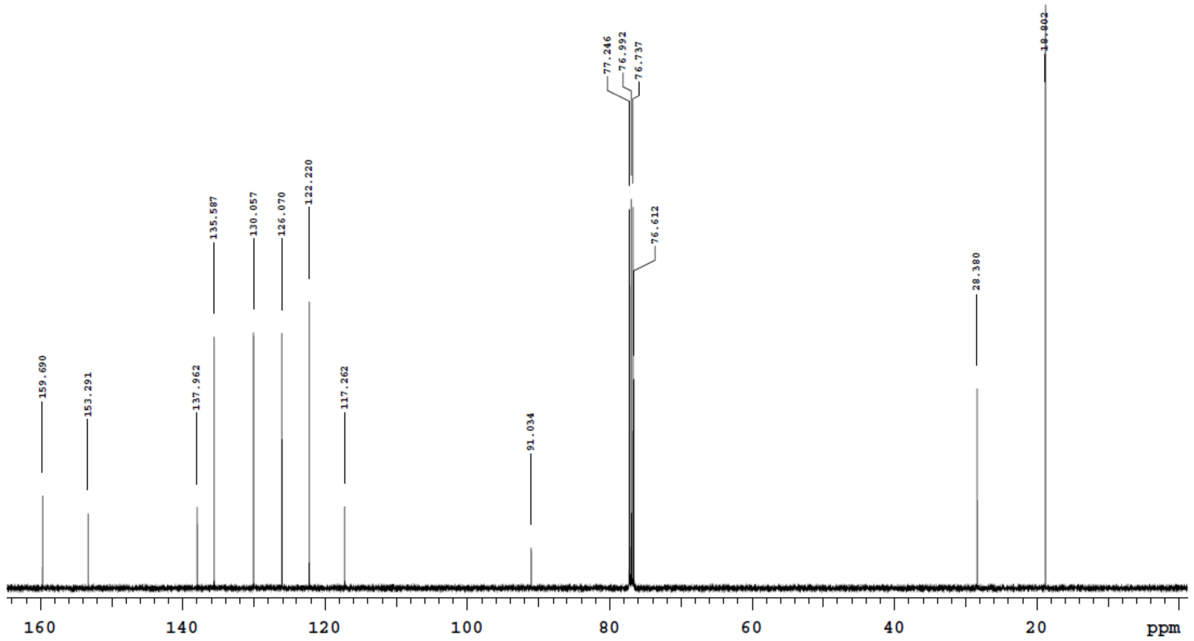

Figure S24. The 125 MHz ^{13}C NMR spectrum of **2f** in $CDCl_3$.

(±)-3-*sec*-Butoxy-4-chloroisocoumarin (**2g**)

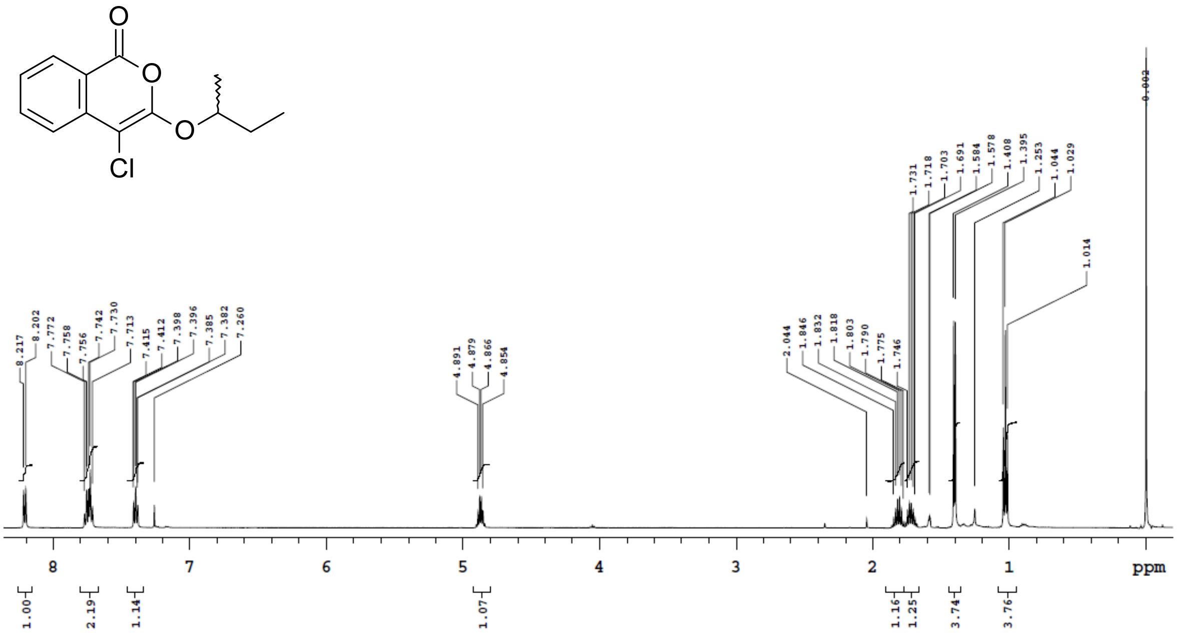

Figure S25. The 500 MHz ^{1}H NMR spectrum of **2g** in $CDCl_3$.

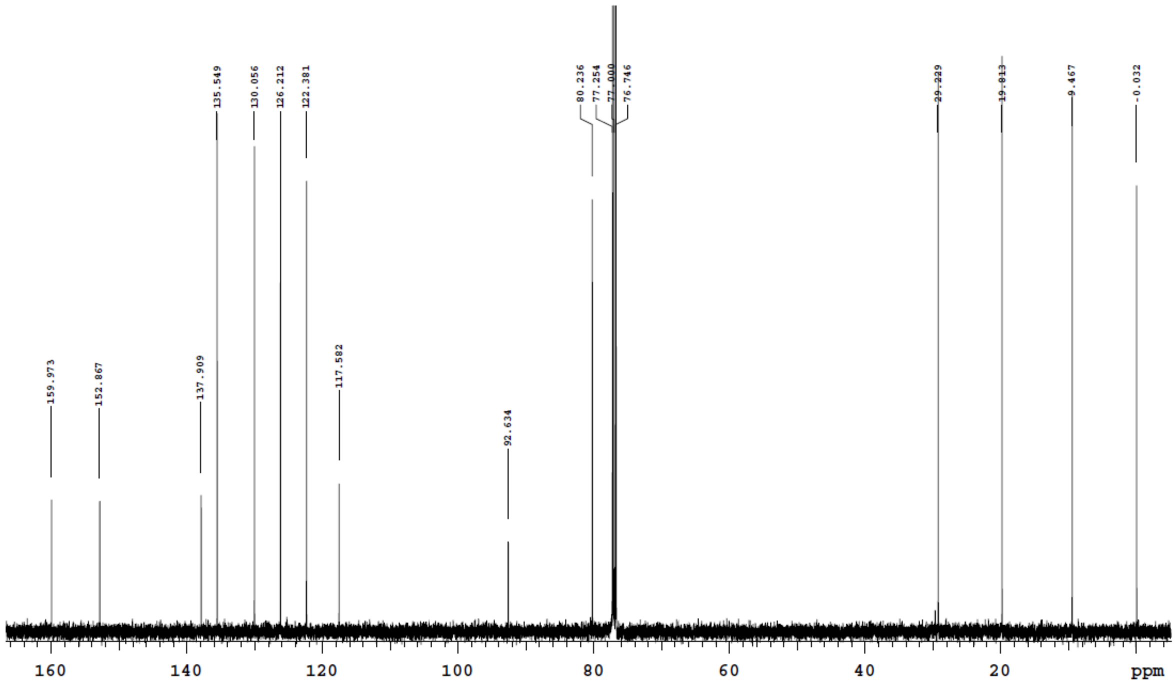

Figure S26. The 125 MHz ^{13}C NMR spectrum of **2g** in $CDCl_3$.

(±)-5-Nitro-2-(2-sec-butoxy-2-oxo-ethyl)benzoic acid (**4**)

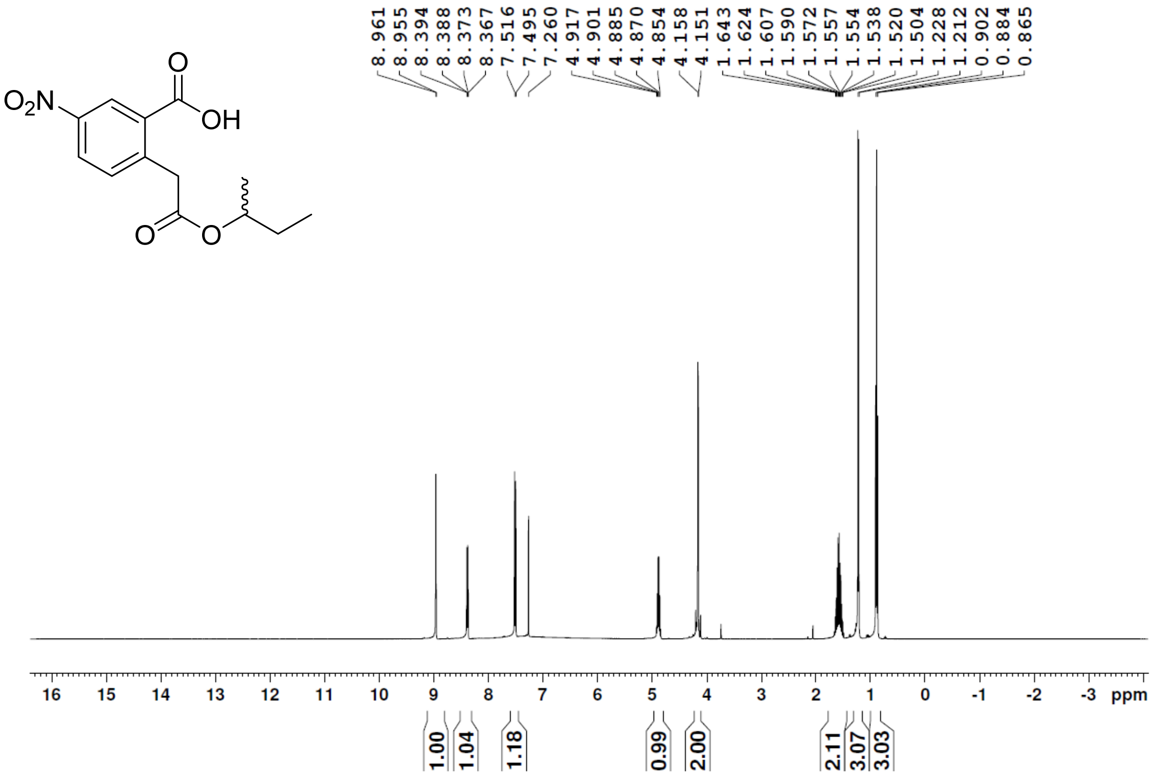

Figure S27. The 500 MHz ¹H NMR spectrum of **4** in CDCl₃.

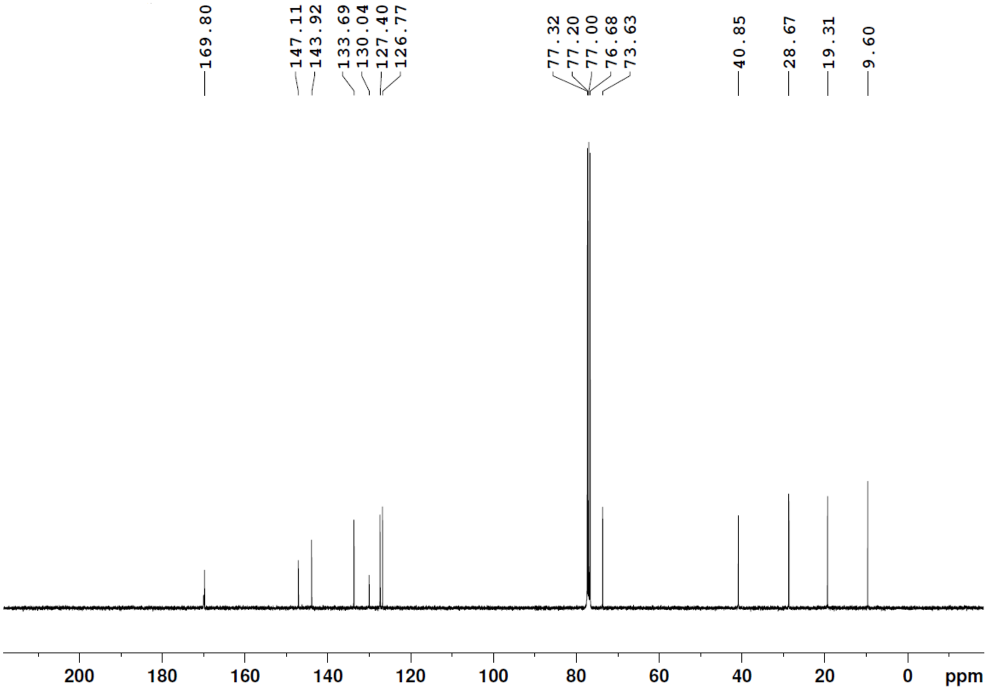

Figure S28. The 125 MHz ¹³C NMR spectrum of **4** in CDCl₃.

(±)-3-*sec*-Butoxy-7-nitro-4-chloroisocoumarin (**5**)

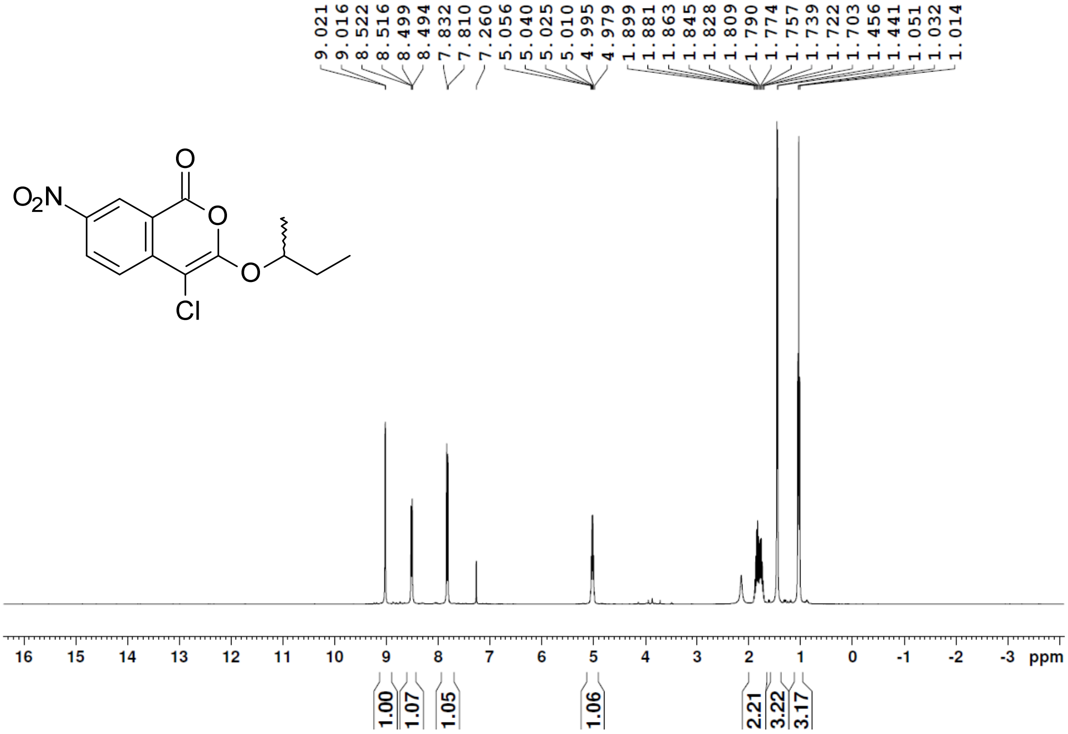

Figure S29. The 400 MHz ^{1}H NMR spectrum of **5** in $CDCl_3$.

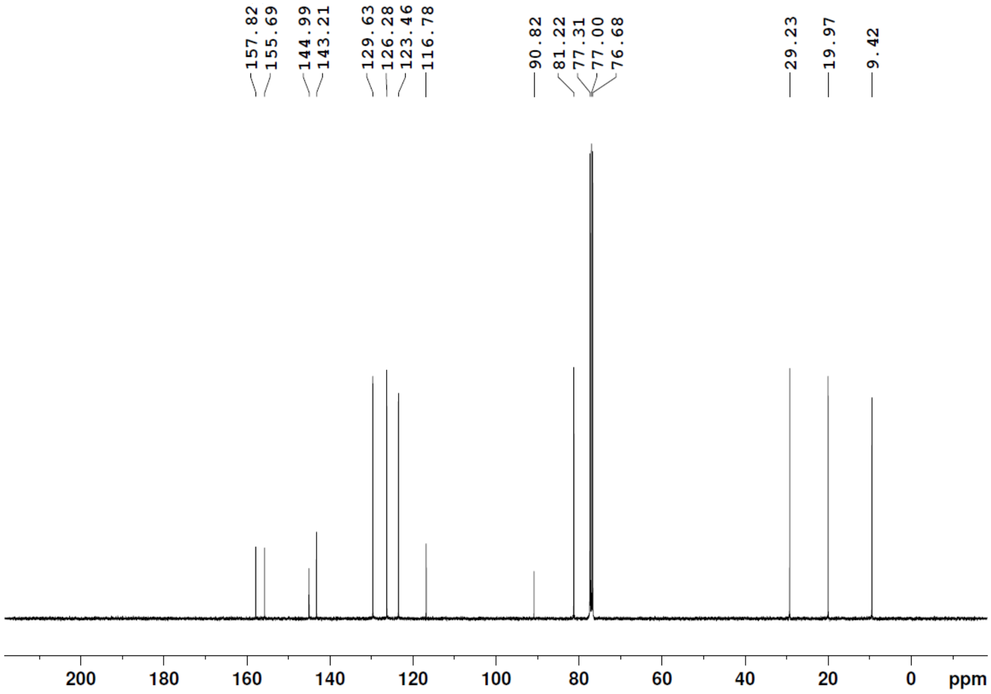

Figure S30. The 125 MHz ^{13}C NMR spectrum of **5** in $CDCl_3$.

(±)-7-Amino-3-*sec*-butoxy-4-chloroisocoumarin (**6a**)

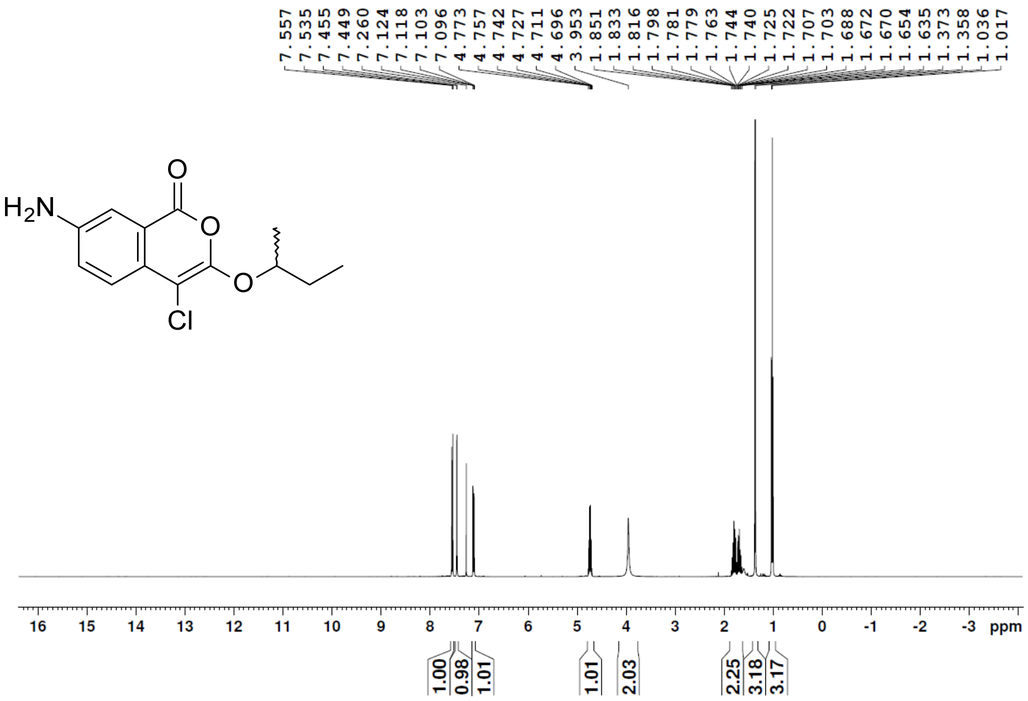

Figure S31. The 400 MHz ^{1}H NMR spectrum of **6a** in $CDCl_3$.

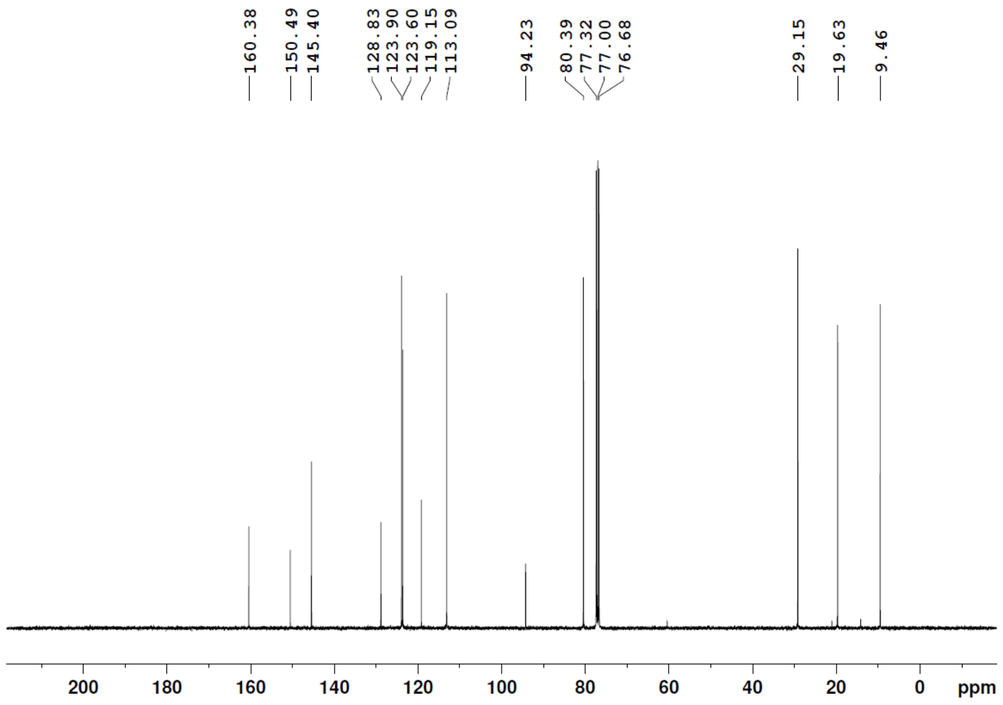

Figure S32. The 100 MHz ^{13}C NMR spectrum of **6a** in $CDCl_3$.

(±)-7-(Dimethylamino)-3-*sec*-butoxy-4-chloroisocoumarin (**6b**)

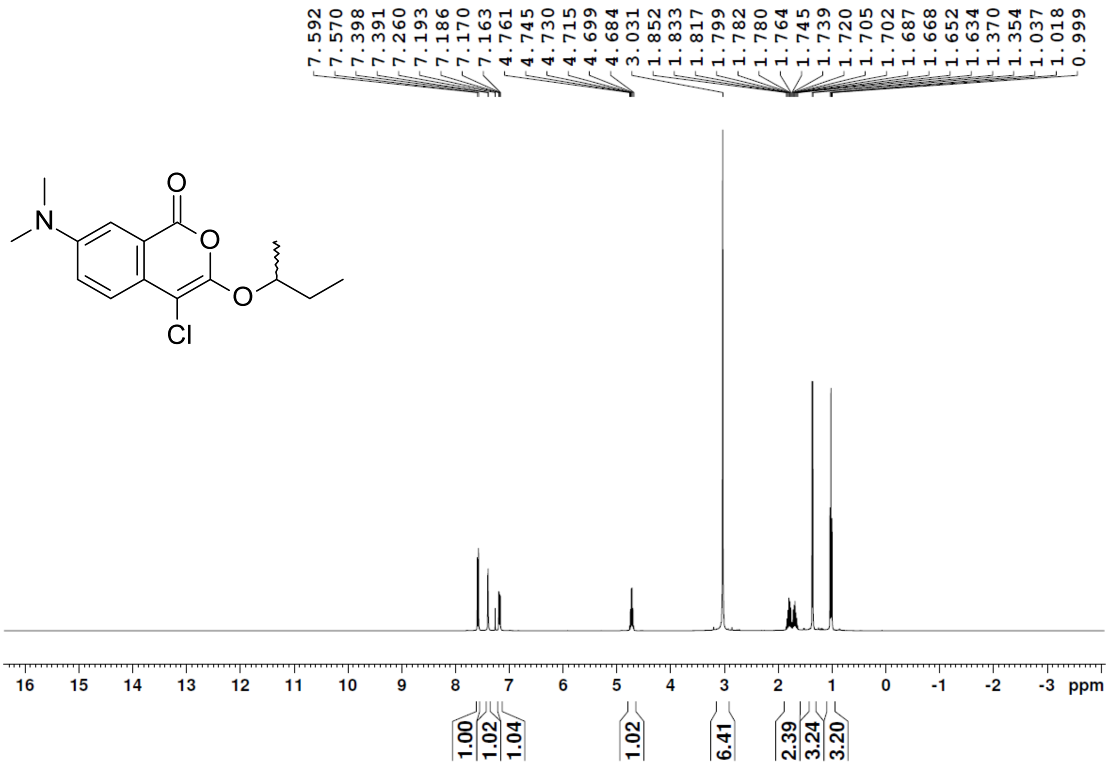

Figure S33. The 400 MHz ^{1}H NMR spectrum of **6b** in $CDCl_3$.

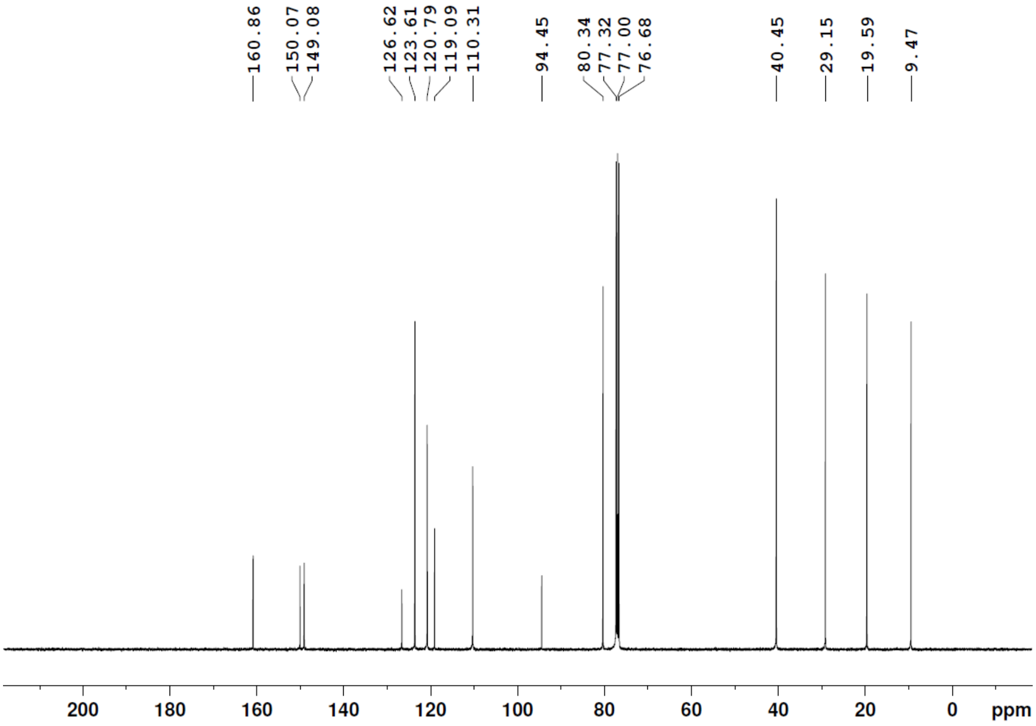

Figure S34. The 100 MHz ^{13}C NMR spectrum of **6b** in $CDCl_3$.

(±)-*N*-(4-Chloro-1-oxo-3-*sec*-butoxy-isochromen-7-yl)acetamide (**6c**)

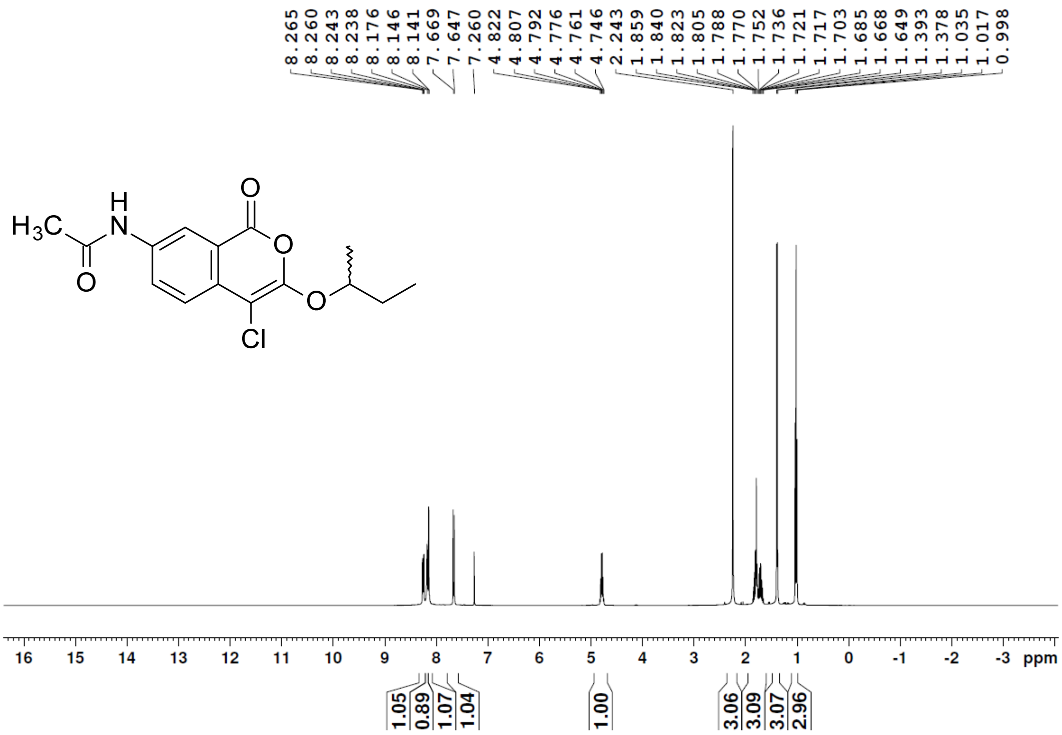

Figure S35. The 400 MHz ^{1}H NMR spectrum of **6c** in $CDCl_3$.

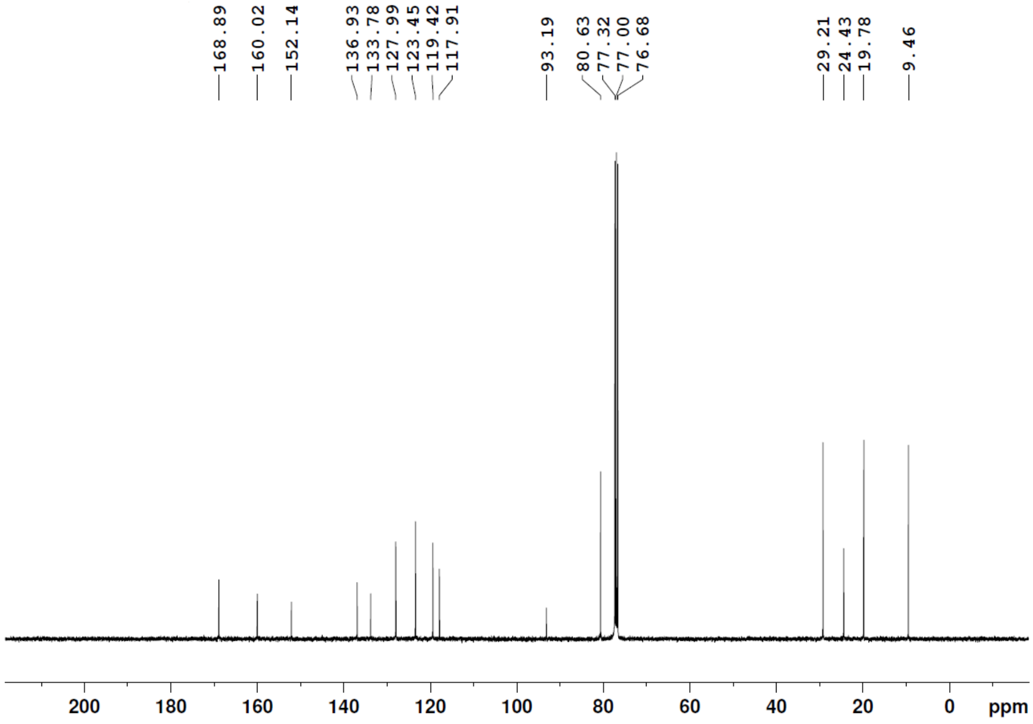

Figure S36. The 100 MHz ^{13}C NMR spectrum of **6c** in $CDCl_3$.

(±)-*N*-(4-Chloro-1-oxo-3-*sec*-butoxy-isochromen-7-yl)-2,2,2-trifluoroacetamide (**6d**)

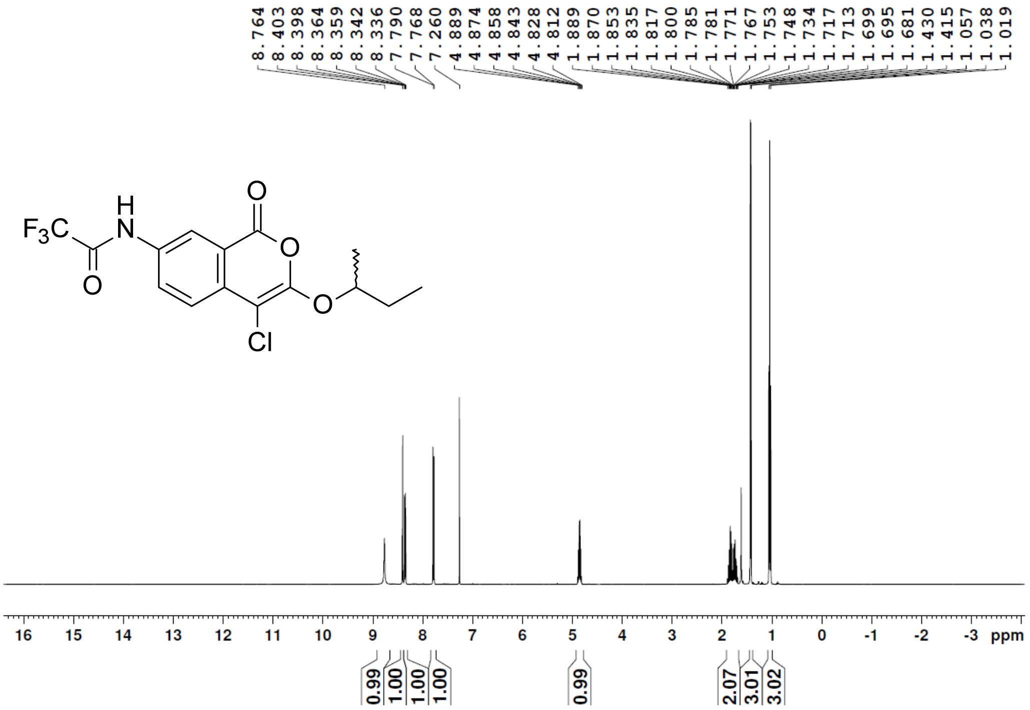

Figure S37. The 400 MHz ^{1}H NMR spectrum of **6d** in $CDCl_3$.

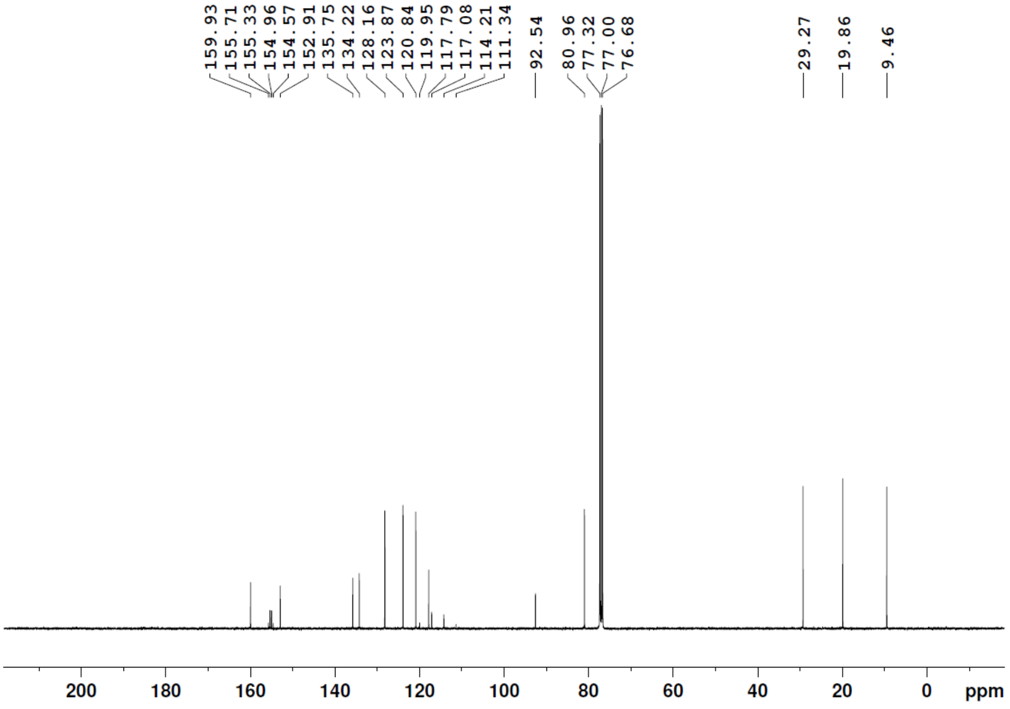

Figure S38. The 100 MHz ^{13}C NMR spectrum of **6d** in $CDCl_3$.

(±)-3-*sec*-Butoxy-4-chloro-7-iodoisocoumarin (**6e**)

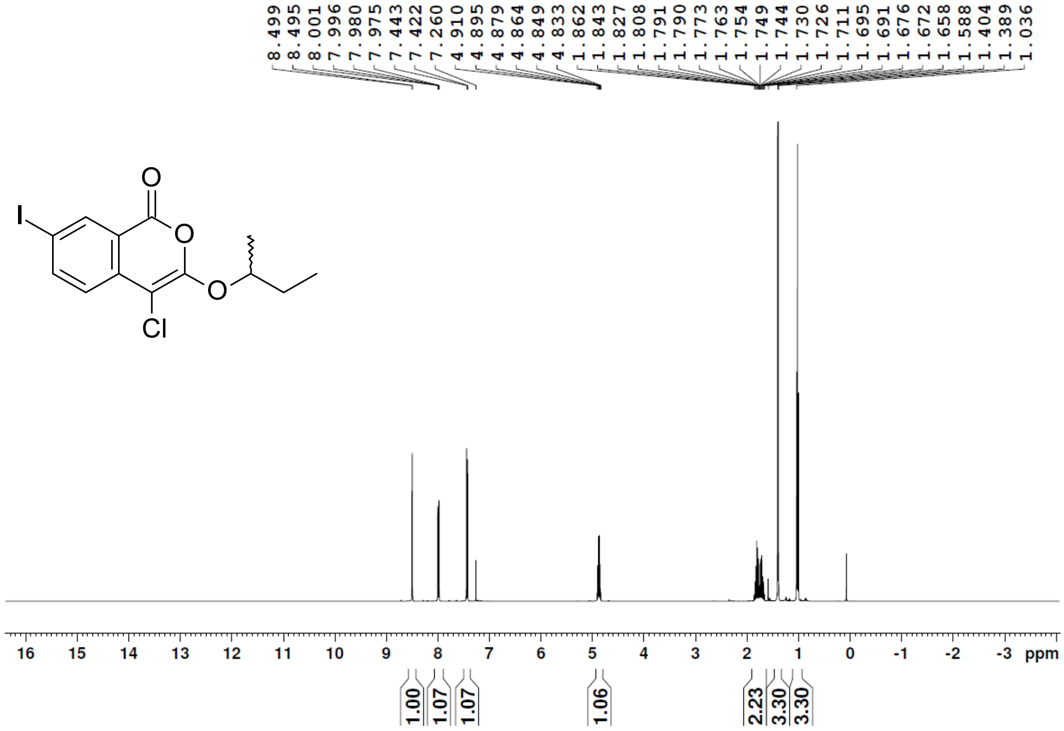

Figure S39. The 400 MHz ^1H NMR spectrum of **6e** in $CDCl_3$.

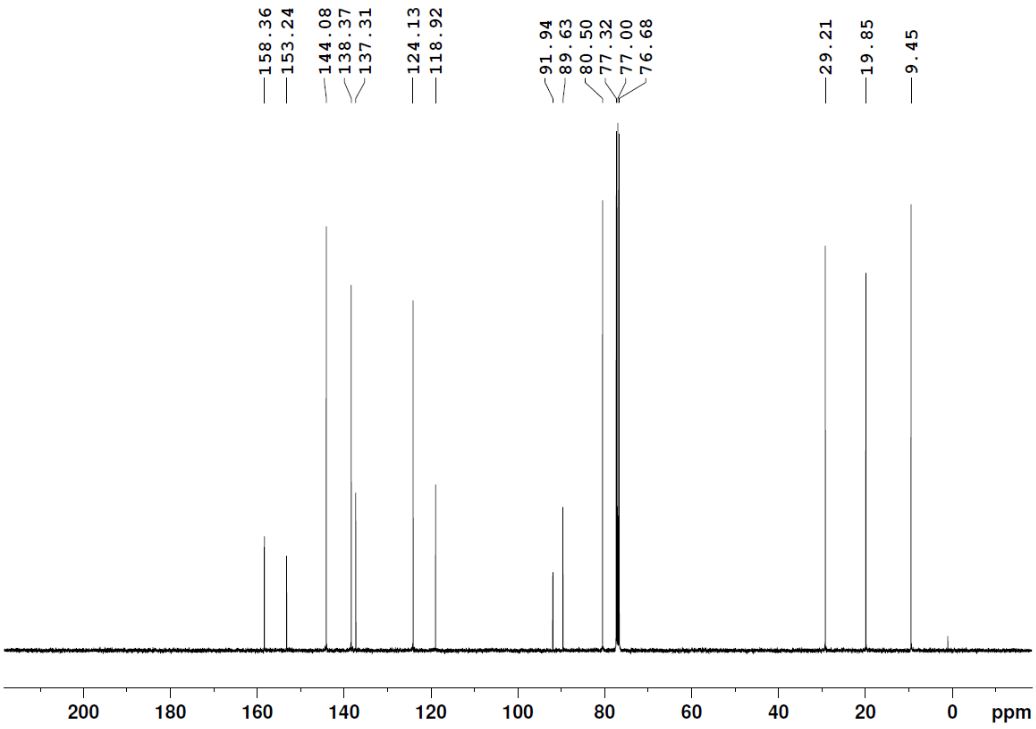

Figure S40. The 100 MHz ^{13}C NMR spectrum of **6e** in $CDCl_3$.

(±)-7-Bromo-3-*sec*-butoxy-4-chloroisocoumarin (**6f**)

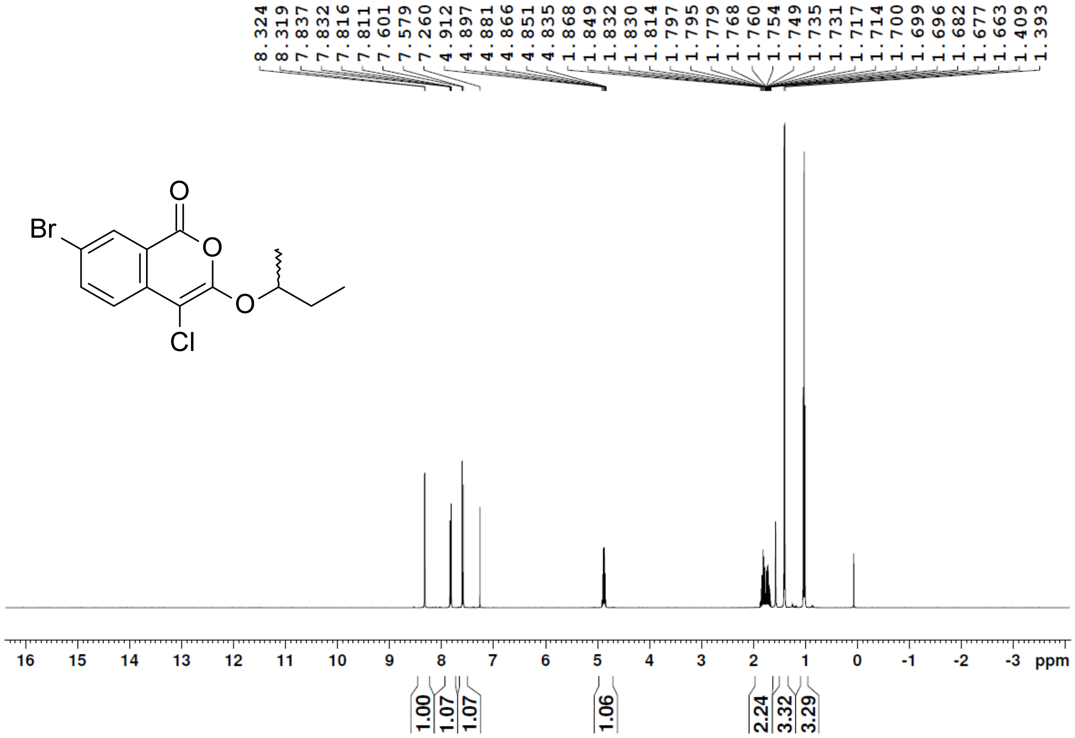

Figure S41. The 400 MHz ^{1}H NMR spectrum of **6f** in $CDCl_3$.

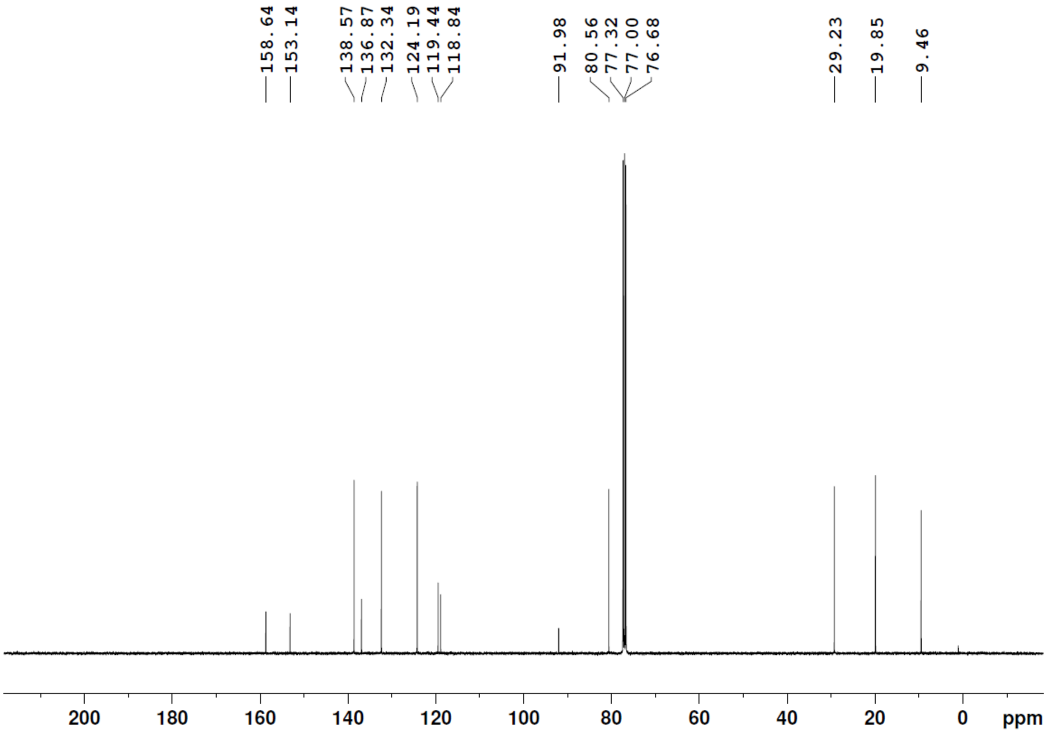

Figure S42. The 100 MHz ^{13}C NMR spectrum of **6f** in $CDCl_3$.

(±)-3-*sec*-Butoxy-4,7-dichloroisocoumarin (**6g**)

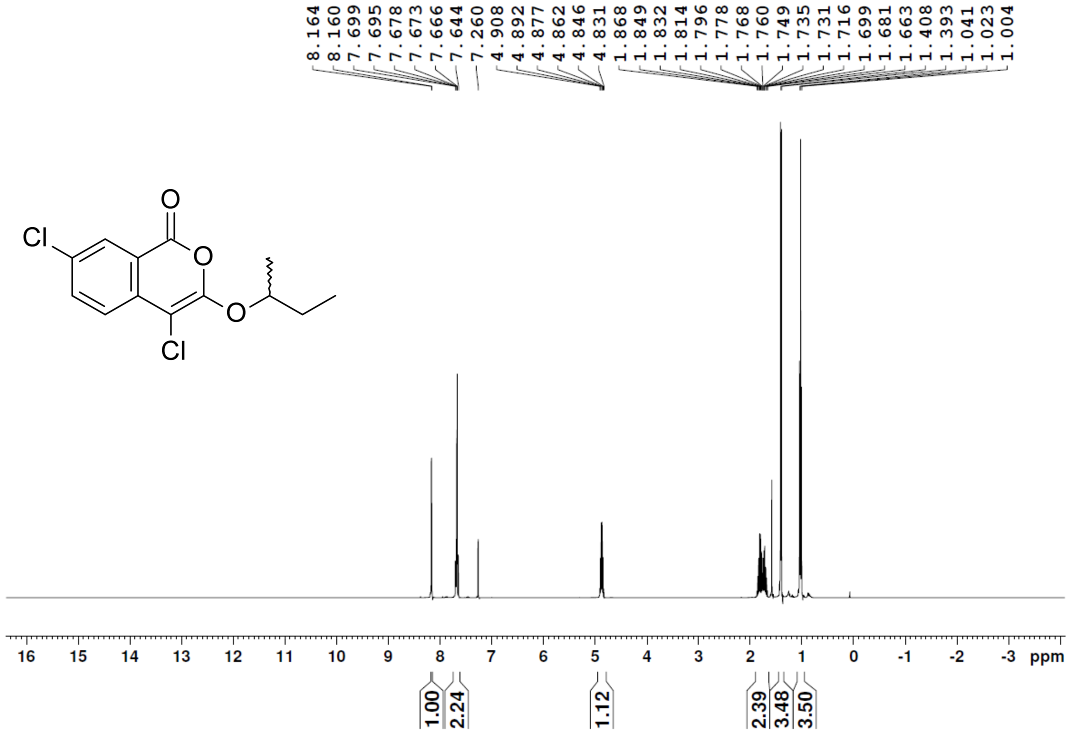

Figure S43. The 400 MHz ^{1}H NMR spectrum of **6g** in $CDCl_3$.

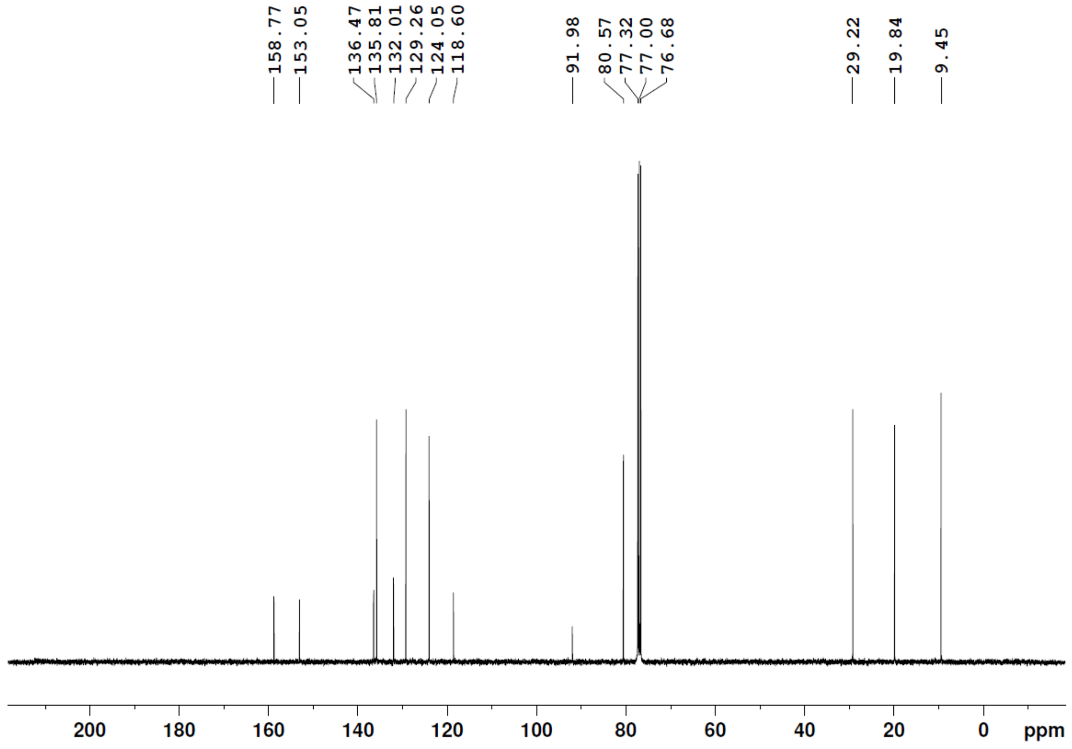

Figure S44. The 100 MHz ^{13}C NMR spectrum of **6g** in $CDCl_3$.

(±)-7-Methoxy-3-*sec*-butoxy-4-chloroisocoumarin (**6h**)

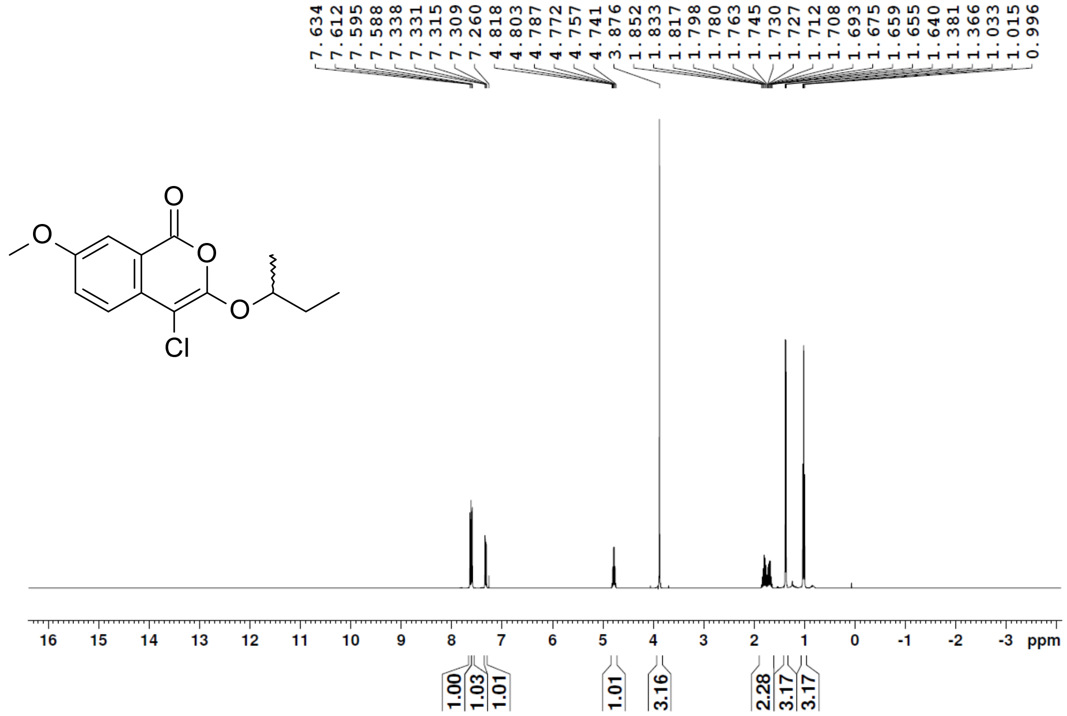

Figure S45. The 400 MHz ^{1}H NMR spectrum of **6h** in $CDCl_3$.

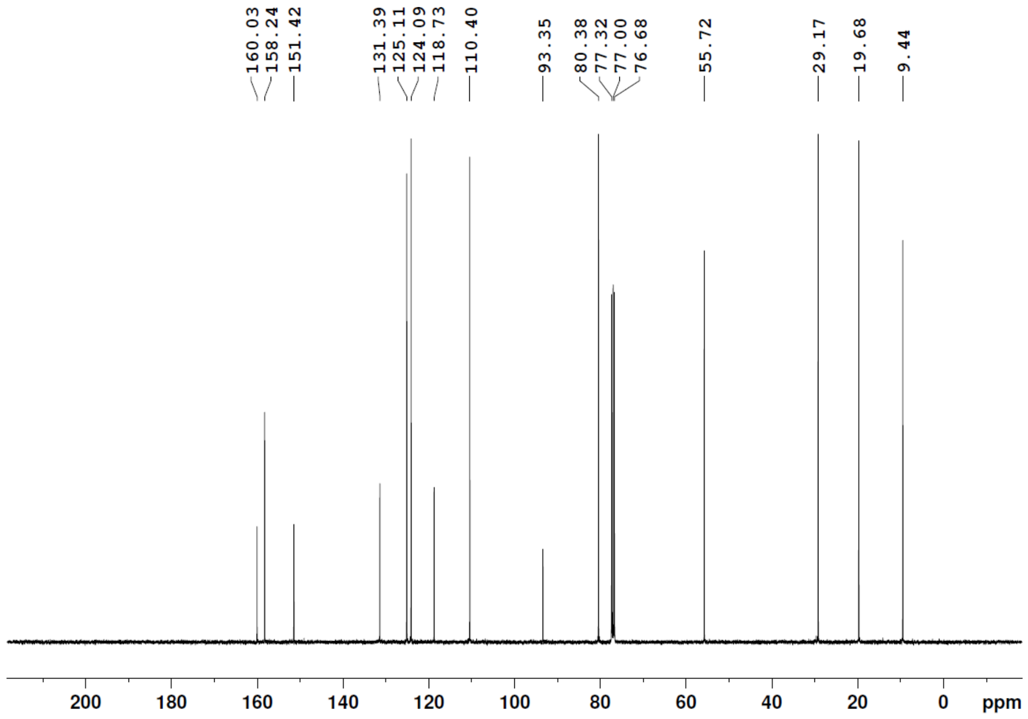

Figure S46. The 100 MHz ^{13}C NMR spectrum of **6h** in $CDCl_3$.

References

References in Supplementary Material follow main text numbering and are listed by order of appearance below:

26. Hansch, C.; Leo, A.; Unger, S.H.; Kim, K.H.; Nikaitani, D.; Lien, E.J. Aromatic substituent constants for structure-activity correlations. *Journal of Medicinal Chemistry* **1973**, *16*, 1207-1216, https://doi.org/10.1021/jm00269a003.

28. Bharti, S.K.; Roy, R. Quantitative 1H NMR spectroscopy. *TrAC Trends in Analytical Chemistry* **2012**, *35*, 5-26, https://doi.org/10.1016/j.trac.2012.02.007.

29. Cushman, M.; Georg, G.I.; Holzgrabe, U.; Wang, S. Absolute Quantitative 1H NMR Spectroscopy for Compound Purity Determination. *Journal of Medicinal Chemistry* **2014**, *57*, 9219-9219, https://doi.org/10.1021/jm501683w.
